# Analysis of the grape (*Vitis vinifera* L.) thaumatin-like protein (TLP) gene family and demonstration that *TLP29* contributes to disease resistance

**DOI:** 10.1038/s41598-017-04105-w

**Published:** 2017-06-27

**Authors:** Xiaoxiao Yan, Hengbo Qiao, Xiuming Zhang, Chunlei Guo, Mengnan Wang, Yuejin Wang, Xiping Wang

**Affiliations:** 1 State Key Laboratory of Crop Stress Biology in Arid Areas, College of Horticulture, Northwest A&F University, Yangling, Shaanxi 712100 China; 2 Key Laboratory of Horticultural Plant Biology and Germplasm Innovation in Northwest China, Ministry of Agriculture, Northwest A&F University, Yangling, Shaanxi 712100 China

## Abstract

Thaumatin-like protein (TLP) is present as a large family in plants, and individual members play different roles in various responses to biotic and abiotic stresses. Here we studied the role of 33 putative grape (*Vitis vinifera* L.) *TLP* genes (*VvTLP*) in grape disease resistance. Heat maps analysis compared the expression profiles of 33 genes in disease resistant and susceptible grape species infected with anthracnose (*Elsinoe ampelina*), powdery mildew (*Erysiphe necator*) or *Botrytis cinerea*. Among these 33 genes, the expression level of *TLP29* increased following the three pathogens inoculations, and its homolog from the disease resistant Chinese wild grape *V*. *quinquangularis* cv. ‘Shang-24’, was focused for functional studies. Over-expression of *TLP29* from grape ‘Shang-24’ (*VqTLP29*) in *Arabidopsis thaliana* enhanced its resistance to powdery mildew and the bacterium *Pseudomonas syringae* pv. tomato DC3000, but decreased resistance to *B*. *cinerea*. Moreover, the stomatal closure immunity response to pathogen associated molecular patterns was strengthened in the transgenic lines. A comparison of the expression profiles of various resistance-related genes after infection with different pathogens indicated that *VqTLP29* may be involved in the salicylic acid and jasmonic acid/ethylene signaling pathways.

## Introduction

Thaumatin is a sweet-tasting protein that was identified in fruits of *Thaumatococcus daniellii* Benth, a plant native to tropical West Africa^1^. It contains a characteristic thaumatin domain common to osmotin-like protein and the PR5-like protein kinase receptor, which are collectively grouped into the thaumatin-like protein (TLP) family^2^. Thaumatin is synthesized first as a precursor protein that is then processed to remove 6 and 22 amino acids at C and N termini, respectively^3^. Most TLP proteins contain the consensus sequence G-x-[GF]-x-C-x-T-[GA]-D-C-x(1,2)-[GQ]-x(2,3)-C^4^. They also contain 16 conserved Cys residues and a REDDD structure, where eight disulfide bonds contribute to maintaining the stability of the protein structure^5^.

TLP proteins are functionally diverse, with proteins from the PR5 subgroup being known for their involvement in biotic and abiotic stress responses^6^, while some TLP genes have been shown to participate in cold, salt and drought stress responses^7–9^. Others are responsible for a broad-spectrum of resistance to multiple pathogens, including *Elsinoe ampelina*, *Verticillium dahliae*, and some filamentous fungi, such as *Botrytis cinerea* and *Fusarium oxysporum* ^10–12^. Thaumatin-like proteins have also been found to combine with G-protein-coupled receptors and their over-expression can confer enhanced resistance to pathogens^13, 14^. Therefore it is needed to understand the role of TLP genes in crop plants in order to enhance disease resistance.

Grape (*Vitis vinifera*) anthracnose (*E*. *ampelina*) and powdery mildew (*E*. *necator*) are some of the most globally widespread fungal diseases^15, 16^. Wild Chinese grape (*V*. *quinquangularis*) exhibits high resistance to a variety of pathogens, and it is an important source of disease resistance genes^16^. The grape *TLP* gene has previously been found to increase host resistance to pathogens^17^, and the grape PR5 protein, VVTL, was reported to inhibit *E*. *ampelina* spore germination and mycelium growth *in vitro* ^18, 19^. It has been shown that the expression of grape *TLP* genes increased after *E*. *ampelina* inoculation, as does the expression of genes encoding a range of antimicrobial proteins, including chitinase and β-1,3 glucanase^20^, PR1/PR1*a* ^21^, stilbene and chalcone synthase^22^, polygalacturonase-inhibitor proteins^23^ and lipid-transfer proteins^24^.

In the current study we extended these earlier studies and more broadly investigated the regulation and potential functions of the grape TLP (*VvTLP*) gene family by evaluating the expression patterns of the different genes in response to different pathogen treatments. These results together with a functional analysis of one *TLP* genes, *VqTLP29*, demonstrated that the grape TLP family is involved in pathogen resistance.

## Results

### Identification of grape *TLP* genes

A total of 33 *TLP* genes were identified in the grape genome sequence. They were named *VvTLP1* to *VvTLP33* based on their distribution and relative linear order on the chromosomes (Table 1). Sixteen of these genes (*VvTLP3*, *VvTLP8*, *VvTLP11*, *VvTLP12*, *VvTLP13*, *VvTLP15*, *VvTLP16*, *VvTLP18*, *VvTLP20*, *VvTLP22*, *VvTLP23*, *VvTLP26*, *VvTLP28*, *VvTLP29*, *VvTLP31* and *VvTLP32*) were predicted to contain both Thaumatin_1 (PS00316) and Thaumatin_2 (PS51367) domain. Eight genes (*VvTLP6*, *VvTLP7*, *VvTLP9*, *VvTLP10*, *VvTLP19*, *VvTLP24*, *VvTLP30* and *VvTLP33*) only contained a Thaumatin_2 domain and 6 genes (*VvTLP1*, *VvTLP2*, *VvTLP14*, *VvTLP17*, *VvTLP25* and *VvTLP27*) had an incomplete Thaumatin_1 structure. A sequence alignment revealed a 9 amino acids difference between the proteins encoded by *VvTLP4* and *VVTL3* in one literature^25^, however since two genes have been assigned to the same chromosomal position, it is likely that they correspond to a single gene. Similarly, *VvTLP10* gene was also confirmed to correspond to *VVTL1* in another literature^18^. Detailed information about each *VvTLP* gene is shown in Table 1.

**Table 1:** Grape *TLP* genes and accession numbers.

Gene ID	Gene locus ID	Accession no.	CDS(bp)	ORF(aa)	Chromosome	Start site	End site	Full length (bp)
*VvTLP1*	GSVIVT01011706001	XM_010656282.1	2451	816	1	4 920 369	4 928 008	7640
*VvTLP2*	GSVIVT01011705001	XM_002284643.2	669	223	1	4 928 289	4 931 588	3300
*VvTLP3*	GSVIVG01019835001	XM_010662912.1	537	178	2	3 754 955	3 774 888	19 934
*VvTLP4*	GSVIVT00001105001	NM_001281202.1	678	225	2	3 757 213	3 758 098	915
*VvTLP5*	GSVIVT00001104001	XM_002276354.3	837	278	2	3 762 619	3 763 455	763
*VvTLP6*	GSVIVG01019836001	XM_002283028.3	333	111	2	3 775 419	3 776 265	847
*VvTLP7*	GSVIVG01019838001	XM_002283006.3	324	108	2	3 790 321	3 791 061	741
*VvTLP8*	GSVIVG01019840001	XM_002282994.2	543	181	2	3 796 865	3 797 720	856
*VvTLP9*	GSVIVG01019841001	XM_002276395.3	276	92	2	3 799 837	3 800 512	676
*VvTLP10*	GSVIVT01019848001	NM_001281132.1	333	110	2	3 821 889	3 822 828	940
*VvTLP11*	GSVIVG01019849001	XM_002282881.2	816	272	2	3 822 830	3 825 957	3128
*VvTLP12*	GSVIVT01024052001	XM_002277426.2	942	314	3	1 484 207	1 486 257	2051
*VvTLP13*	GSVIVG01024050001	XM_002277512.3	1050	350	3	1 492 908	1 494 948	2041
*VvTLP14*	GSVIVT00008703001	XM_010651172.1	252	84	4	16 284 385	16 288 803	639
*VvTLP15*	GSVIVG01018769001	XM_002265889.3	891	297	4	20 085 218	20 086 815	1598
*VvTLP16*	GSVIVG01018767001	XM_002265769.2	1008	336	4	20 090 880	20 092 677	1798
*VvTLP17*	GSVIVT01024997001	XM_002281157.3	609	203	6	5 401 162	5 404 988	3827
*VvTLP18*	GSVIVT01034131001	XM_002271081.3	2832	943	8	14 959 909	14 971 330	11 422
*VvTLP19*	GSVIVG01033694001	XM_002265816.2	729	242	8	18 591 043	18 591 956	914
*VvTLP20*	GSVIVT01022993001	XM_002273235.3	870	316	12	17 212 234	17 213 401	1168
*VvTLP21*	GSVIVT00008847001	XM_002264514.2	510	169	13	14 942 681	14 943 190	1526
*VvTLP22*	GSVIVG01032051001	XM_002264514.2	630	210	13	23 191 363	23 192 223	861
*VvTLP23*	GSVIVT01016504001	XM_002278043.2	735	244	13	3 332 382	3 333 610	1229
*VvTLP24*	GSVIVG01032560001	XM_010662681.1	618	206	14	28 370 966	28 373 424	2459
*VvTLP25*	GSVIVT01018147001	AM458348.2	204	68	15	13 903 826	13 904 404	579
*VvTLP26*	GSVIVG01027712001	XM_010663383.1	573	191	15	13 966 172	13 966 744	573
*VvTLP27*	GSVIVT01027698001	XM_002269236.3	246	82	15	14 170 030	14 194 374	24 345
*VvTLP28*	GSVIVG01038679001	XM_010664644.1	888	296	16	21 050 289	21 051 593	1305
*VvTLP29*	GSVIVG01008423001	XM_003634158.2	735	245	17	2 254 244	2 255 631	1388
*VvTLP30*	GSVIVG01009646001	NM_001281159.1	525	175	18	10 174 163	10 175 673	1511
*VvTLP31*	GSVIVG01009928001	XM_002274101.3	951	317	18	12 468 309	12 470 528	2220
*VvTLP32*	GSVIVG01009930001	XM_010666529.1	948	316	18	12 488 625	12 490 747	2123
*VvTLP33*	GSVIVT01008918001	XM_010656282.1	927	308	18	3 387 828	3 389 344	1517

CDS: coding sequence, ORF: open reading frame.

### Phylogenetic analysis and exon-intron organization

**Figure 1:**
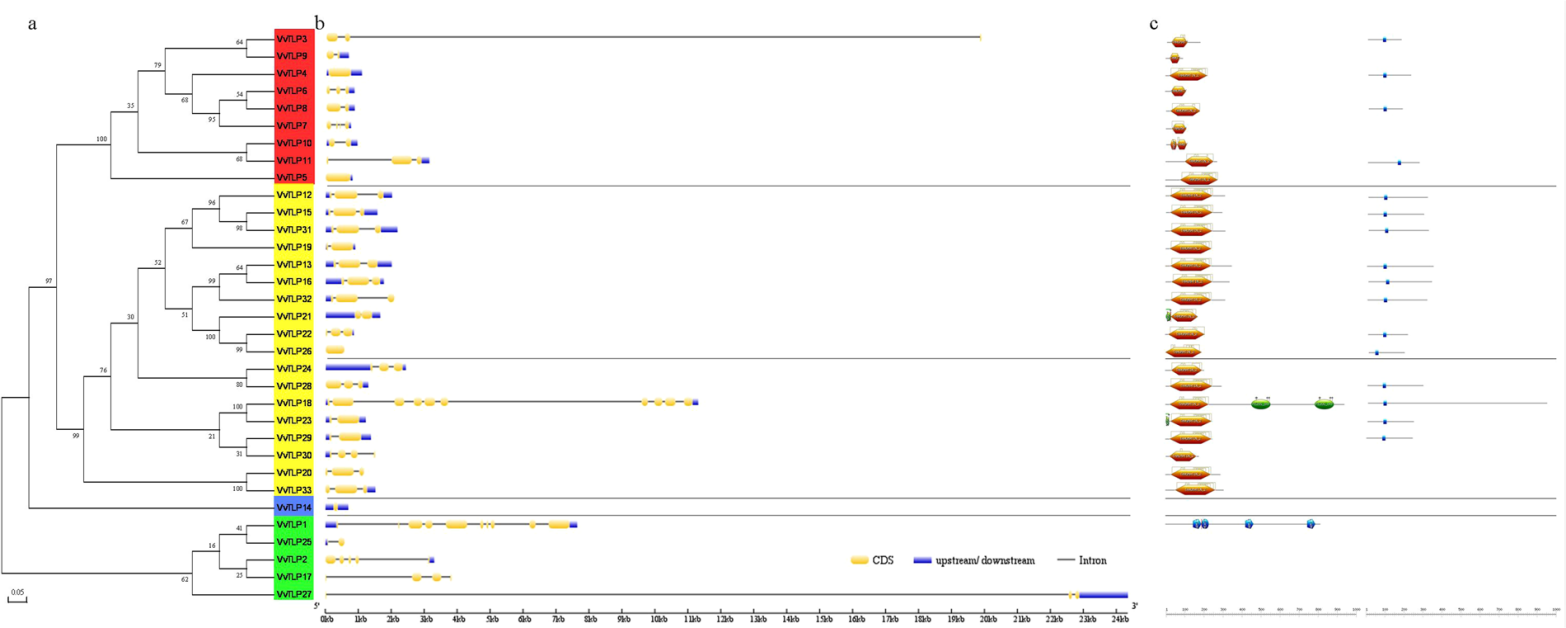
Genome wide organization of grape TLP (*VvTLP*) genes. (**a**) Phylogenetic tree based on the protein sequences of 33 *VvTLP* genes. Phylogenetic tree was constructed using the neighbor-joining method with MEGA5. Four subfamilies (Type I, II, III and IV) were analyzed and colored in red, yellow, blue and green, respectively. Bootstrap values at the nodes from 1000 replicates were used to assess the robustness of the tree. The scale is in amino acid substitutions per site. (**b**) Exon-intron structure of *VvTLP* genes: yellow indicates coding sequence (CDS), blue indicates untranslated 5′- and 3′- regions, black indicates introns. (**c**) Structures of *VvTLP* proteins: brown indicates Thaumatin_2 domain, blue rectangle indicates Thaumatin_1 domain, green ellipse in VvTLP21 and VvTLP23 indicates a PROKAR-lipoprotein domain, green ellipse in VvTLP18 indicates a FE2OG_OXY domain, blue pentagon indicates an ARM_REPEAT domain, blue ellipse indicates a HEAT_REPEAT domain, gray line indicates a disulfide bridge, gray icon indicates active sites, green line indicates undefined bridge/range.

A phylogenetic tree was constructed using the protein sequences of all the 33 *VvTLP* genes. The grape *VvTLP* family was divided into 4 subfamilies (Type I, II, III and IV, Fig. 1a). The Type II subfamily colored in yellow (54.55%) contained the most members, followed by Type I colored in red (27.27%) and Type IV colored in green (15.15%). The least represented subfamily was Type III colored in blue (3.03%) with only *VvTLP14*. Besides, thirty-one *VvTLP* genes shown in gene structure analysis had less than 4 exons, while *VvTLP1* had 9 and *VvTLP18* had 10 exons. Four genes (*VvTLP4*, *VvTLP5*, *VvTLP14* and *VvTLP26*) had no introns (Fig. 1b). Analysis of the protein domain organization showed that the thaumatin domain was present in 27 of the 33 grape *TLP* genes from Type I and II subfamily (Fig. 1c). In addition, *VvTLP1* was predicted to contain three ARM_REPEAT (Armadillo/plakoglobin repeat) domains functioned as the cell-contact and cytoskeleton-associated proteins^26^, and a HEAT_REPEAT domain associated with chromosome dynamics and functions, including the transcription factors and microtubule-associated proteins^27^. *VvTLP18* contained two FE2OG_OXY (Fe^2+^ 2-oxoglutarate dioxygenase) domains involved in the oxidation of the organic substrate using a dioxygen molecule^28^. While both *VvTLP21* and *VvTLP23* had a PROKAR (Prokaryotic membrane lipoprotein lipid attachment) domain functioned as the signal peptidase^29^.

### Tandem duplication and synteny analysis

**Figure 2:**
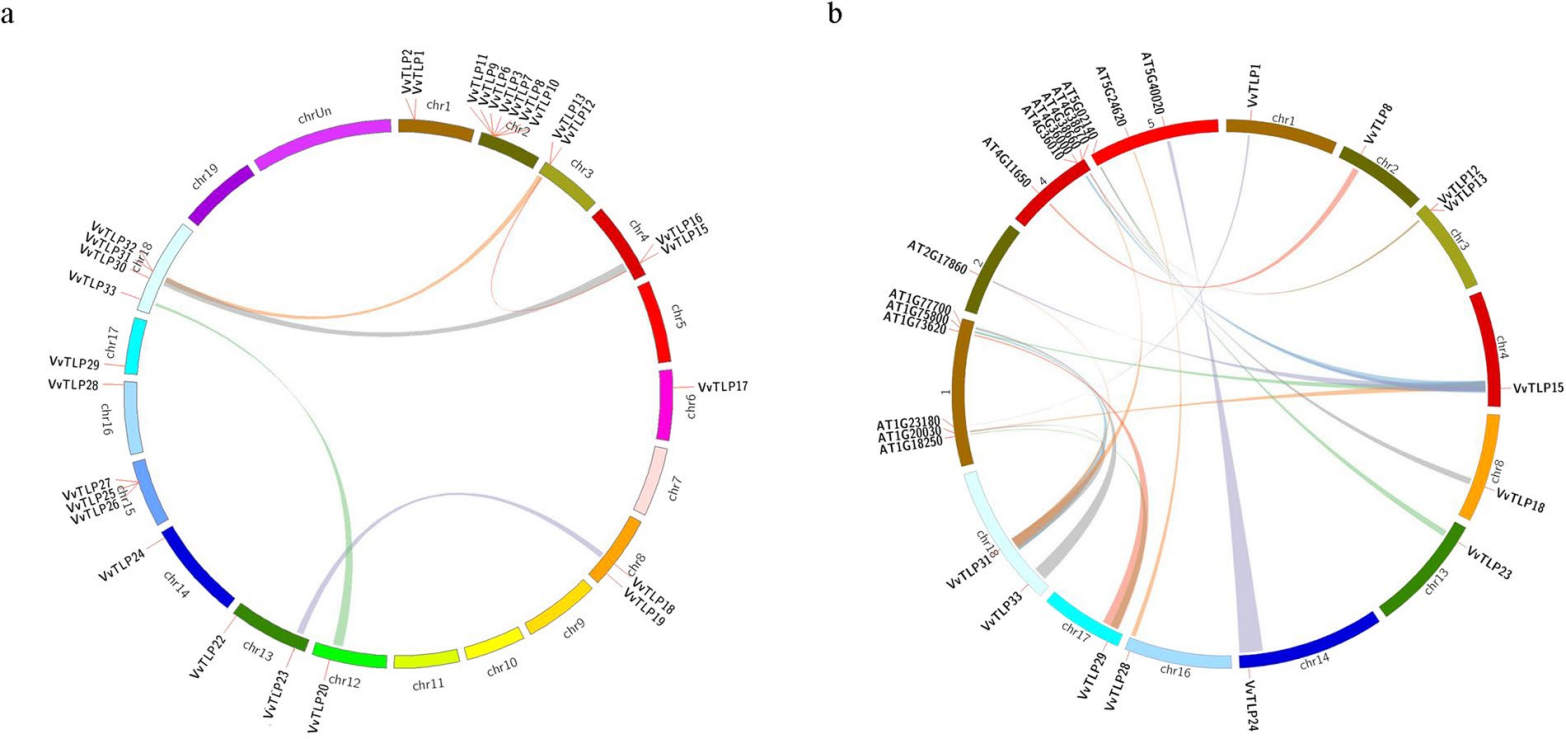
Chromosome distribution and synteny analysis of grape and *Arabidopsis thaliana TLP* genes. (**a**) Chromosomes 1–19 are shown in different colors in a circular diagram. The approximate distribution of each *VvTLP* gene is marked with a short red line on the circle. Colored curves denote the details of syntenic regions between grape *TLP* genes. (**b**) The chromosomes of grape and *A*. *thaliana* are depicted as a circle. The approximate distribution of each *AtTLP* and *VvTLP* gene is marked with a short red line on the circle. Colored curves denote the details of syntenic regions between grape and *A*. *thaliana TLP* genes.

Tandem duplication events associated with the 29 thaumatin domain containing *VvTLP* genes were analyzed next except for four genes (*VvTLP4*, *VvTLP5*, *VvTLP14* and *VvTLP21*). A total of 18 genes (*VvTLP1*, *VvTLP2*, *VvTLP3*, *VvTLP6*, *VvTLP7*, *VvTLP8*, *VvTLP9*, *VvTLP10*, *VvTLP11*, *VvTLP12*, *VvTLP13*, *VvTLP15*, *VvTLP16*, *VvTLP25*, *VvTLP26*, *VvTLP27*, *VvTLP31* and *VvTLP32*) clustered into 6 tandem duplication event regions on grape chromosome 1, 2, 3, 4, 15 and 18, indicating that more than half of the *VvTLP* genes were generated by gene duplication (Fig. 2a). Tandem duplication was also found between *VvTLP18* and *VvTLP23*, *VvTLP20* and *VvTLP33*, but was not shown in 7 genes (*VvTLP17*, *VvTLP19*, *VvTLP22*, *VvTLP24*, *VvTLP28*, *VvTLP29* and *VvTLP30*). A synteny analysis of the *Arabidopsis thaliana* and grape *TLP* genes further revealed the 20 syntenic relations that contain 15 *AtTLP* genes and 12 *VvTLP* genes (Fig. 2b).

### *VvTLP* expression profiles

**Figure 3:**
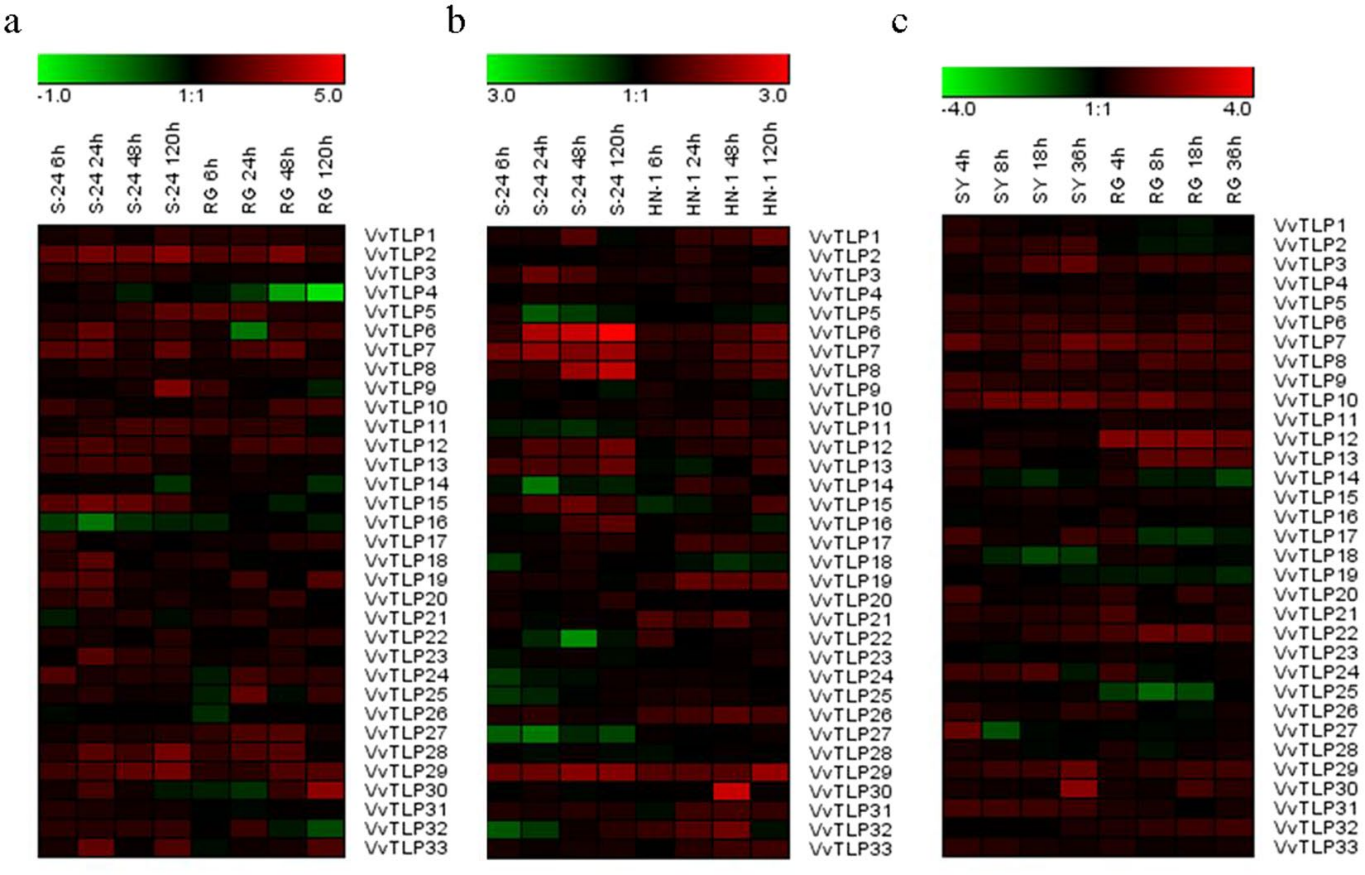
Expression profiles of 33 *VvTLP* genes. The expression data from the semi-quantitative RT-PCR analyses were analyzed and visualized into heat maps using the Gene Tools software and MeV 4.8.1. The color scale represents relative expression levels, with red and green indicating increased or decreased transcript abundance, respectively. (**a**) Expression profiles of *VvTLP* genes following anthracnose (*E*. *ampelina*) inoculation (6, 24, 48 and 120 h) in both ‘Shang-24’ and Red Globe. (**b**) Expression profiles of *VvTLP* genes after powdery mildew (*E*. *necator*) inoculation (6, 24, 48 and 120 h) in both ‘Shang-24’ and ‘Hunan-1’. (**c**) Expression profiles of *VvTLP* genes in response to *B*. *cinerea* inoculation (4, 8, 18 and 36 h) in both ‘Shuangyou’ and Red Globe. ‘S-24’, ‘RG’, ‘HN-1’ and ‘SY’ represent names of corresponding grape varieties. Original results are shown in Supplementary Fig. S1. The experiments were repeated three times and the results were found consistent.

We conducted a systematic expression analysis of all the 33 *VvTLP* genes in grape plants following inoculation with three pathogens. Anthracnose (*E*. *ampelina*), powdery mildew (*E*. *necator*) and *B*. *cinerea* were used to infect the anthracnose-resistant grape ‘Shang-24’ (*V*. *quinquangularis*) and anthracnose-susceptible grape Red Globe (*V*. *vinifera*)^15, 30^, powdery mildew-resistant grape ‘Shang-24’ and powdery mildew-susceptible grape ‘Hunan-1’ (*V*. *pseudoreticulata*)^16^, and *B*. *cinerea*-resistant ‘Shuangyou’ (*V*. *amurensis*) and *B*. *cinerea*-susceptible grape Red Globe^31^, respectively. The heat maps of resulting expression profiles were shown in Fig. 3 and the semi-quantitative RT-PCR and real-time quantitative PCR expression data were shown in Supplementary Figure S1. The expression levels of 23 genes (*VvTLP2*, *VvTLP3*, *VvTLP5*, *VvTLP6*, *VvTLP7*, *VvTLP8*, *VvTLP9*, *VvTLP10*, *VvTLP11*, *VvTLP12*, *VvTLP13*, *VvTLP15*, *VvTLP18*, *VvTLP19*, *VvTLP22*, *VvTLP23*, *VvTLP24*, *VvTLP25*, *VvTLP27*, *VvTLP28*, *VvTLP29*, *VvTLP31* and *VvTLP33*) increased following the anthracnose inoculation. The expression levels of 14 genes (*VvTLP1*, *VvTLP3*, *VvTLP6*, *VvTLP7*, *VvTLP8*, *VvTLP12*, *VvTLP13*, *VvTLP15*, *VvTLP16*, *VvTLP17*, *VvTLP19*, *VvTLP20*, *VvTLP26* and *VvTLP29*) increased following the powdery mildew inoculation. And the expression levels of 19 genes (*VvTLP2*, *VvTLP3*, *VvTLP5*, *VvTLP6*, *VvTLP7*, *VvTLP8*, *VvTLP9*, *VvTLP10*, *VvTLP12*, *VvTLP17*, *VvTLP20*, *VvTLP21*, *VvTLP22*, *VvTLP24*, *VvTLP26*, *VvTLP29*, *VvTLP30*, *VvTLP31* and *VvTLP33*) increased following the *B*. *cinerea* inoculation. Among these genes, the expression levels of 6 genes (*VvTLP3*, *VvTLP6*, *VvTLP7*, *VvTLP8*, *VvTLP12* and *VvTLP29*) were simultaneously up-regulated following the three pathogens inoculations.

### Response of *VqTLP29* over-expressing *A*. *thaliana* lines to powdery mildew (*Golovinomyces cichoracearum* UCSC1) challenge

**Figure 4:**
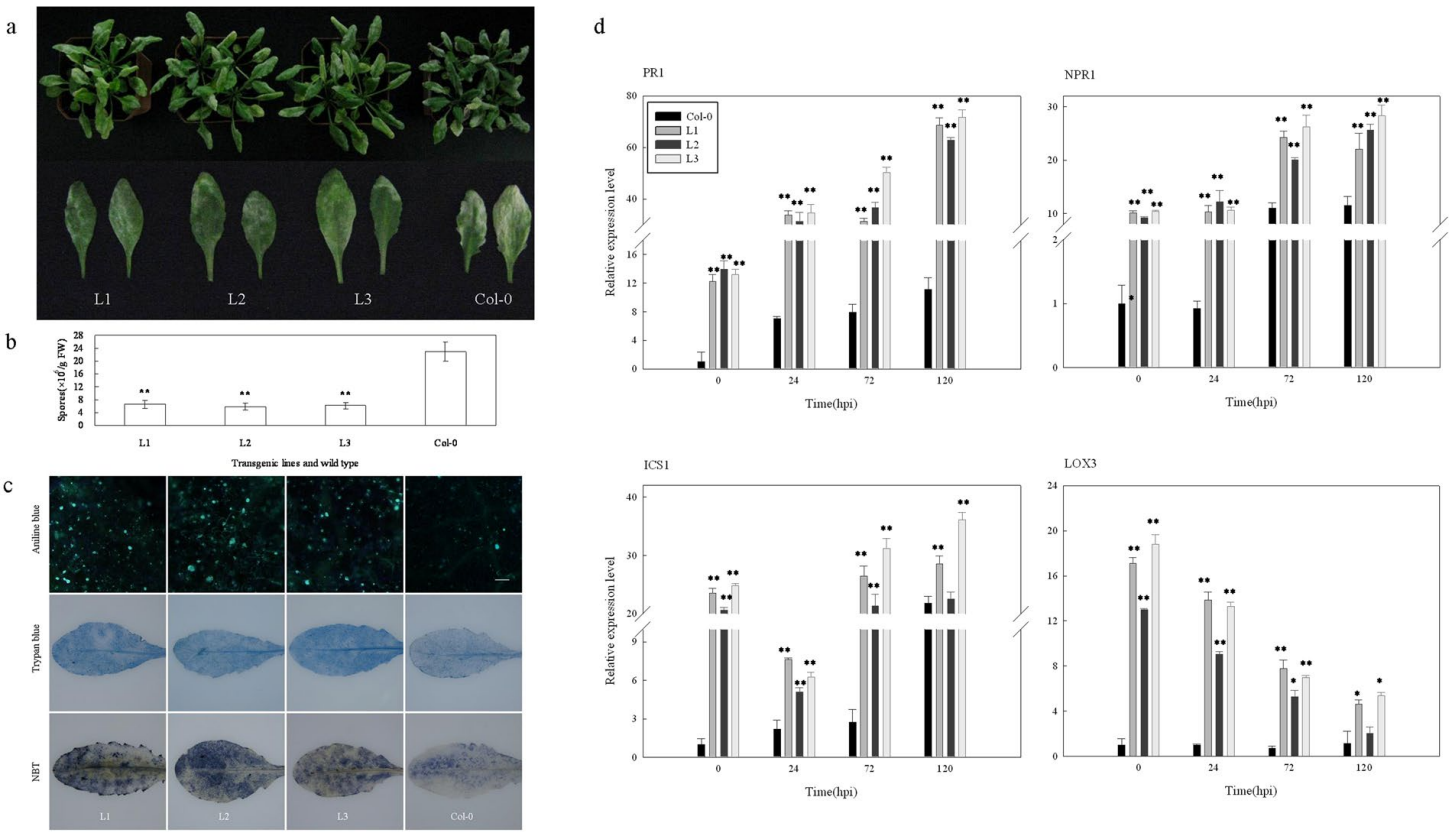
Response of *VqTLP29* over-expressing *Arabidopsis thaliana* lines to powdery mildew inoculation. (**a**) Phenotypes of the 4-week old *VqTLP29* transgenic line L1, L2, L3 and Col-0 infected with powdery mildew and disease symptoms in representative rosettes after inoculation for 7 days. (**b**) The number of spores per gram fresh leaf 7 days post powdery mildew inoculation. Spore suspensions of the *VqTLP29* transgenic lines and Col-0 were diluted in sterile water with 10 diseased leaves and counted using the hemocytometer. Data were combined and analyzed as a one-way ANOVA in three independent experiments. Asterisks indicate statistical significance (**P < 0.01). (**c**) Callose deposition, cell death and O^2−^ accumulation in the *VqTLP29* transgenic lines and Col-0 7 days post powdery mildew inoculation. Three infected leaves of the transgenic lines and Col-0 were required for each stain in three independent experiments. The scale bar in the figure showing aniline blue staining indicates 100 μm. (**d**) Expression levels of disease resistance genes in the *VqTLP29* transgenic lines and Col-0 at 0, 24, 72 and 120 hpi following powdery mildew inoculation. Asterisks indicate statistical significance (*0.01 < P < 0.05, **P < 0.01, one-way ANOVA). The experiments were repeated three times with consistent results.

Based on its strong induction by pathogen infection (Fig. 3), *VqTLP29* was selected for further functional analysis by constitutive over-expression in *A*. *thaliana*. Sequence homology between TLP29 from ‘Shang-24’ and Red Globe and the analysis of the thaumatin domain in all TLP29 proteins were shown in Supplementary Datas S1 and S2. Three verified transgenic *A*. *thaliana* line L1, L2 and L3 were inoculated with the causal agent of powdery mildew, *Golovinomyces cichoracearum* UCSC1, and were found with enhanced resistance, compared to wild type Col-0 at 7 days post-inoculation (Fig. 4a). Spores were eluted from infected leaves of the transgenic lines and Col-0, counted and significantly lower concentrations of spores were observed from the transgenic lines (Fig. 4b). A histochemical staining assay showed that callose deposition in all three transgenic lines was more widely distributed than in Col-0 (Fig. 4c), and the frequency of cell death and O^2−^ levels were both higher in the transgenic lines. We also examined the expression of key genes known to be involved in hormone synthesis or signal transduction pathways in the *VqTLP29* over-expressing lines and Col-0. These included *PR1* and *NPR1*, which are involved in salicylic acid (SA) signaling pathway, *NPR1*, a central regulator gene in the SA signaling pathway^32^, *ICS1* and *LOX3*, which are integrant for both SA and jasmonic acid (JA) biosynthesis^33, 34^. The expression levels of *VqTLP29* following *G*. *cichoracearum* inoculation increased in the three transgenic lines (Supplementary Figure S2a), and the expression levels of *PR1*, *NPR1* and *ICS1* were also found to be higher in the transgenic lines, peaking at 120 hpi (hours post inoculation). The expression levels of *LOX3* gradually decreased to the lowest level at 120 hpi in the transgenic lines (Fig. 4d).

### Response of *VqTLP29* over-expressing *A*. *thaliana* lines to *B*. *cinerea* inoculation

**Figure 5:**
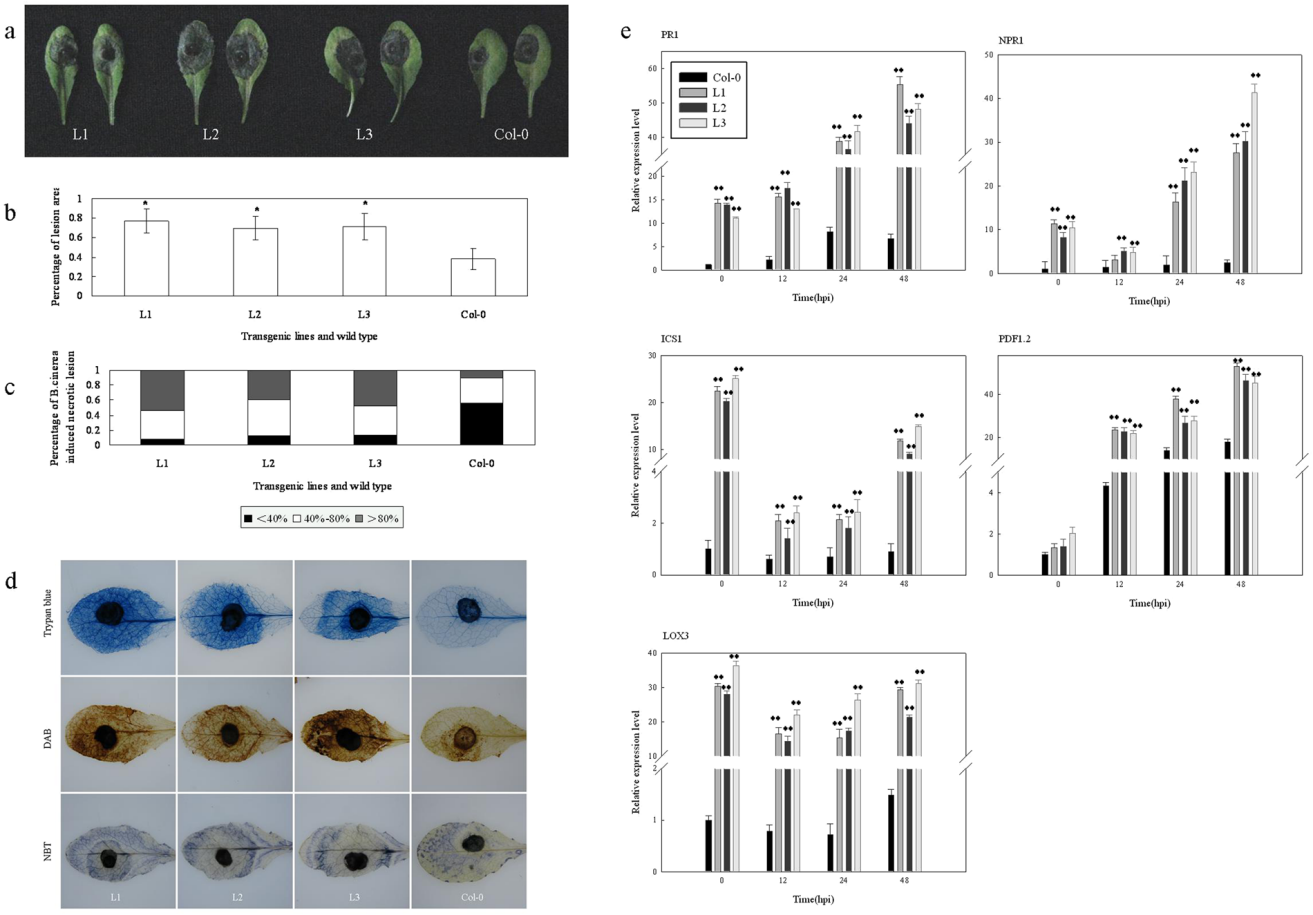
Response of *VqTLP29* over-expressing *Arabidopsis thaliana* lines to *B*. *cinerea* inoculation. (**a**) Disease symptoms in representative rosettes of the 4-week old *VqTLP29* transgenic line L1, L2, L3 and Col-0 after *B*. *cinerea* inoculation for 3 days. Leaves were detached and inoculated by dropping 10 μl spore suspension with the concentration of 2.0 × 10^6^ spores ml^−1^ onto the adaxial surface. Leaves were highly moisturizing until the lesion shown. (**b**) Percentage of the leaves covered by the lesions 3 days post *B*. *cinerea* inoculation. Lesion sizes of the *VqTLP29* transgenic lines and Col-0 were measured in three independent experiments using grid statistics. Data represent mean values ± SD with 50 leaves per sample. Asterisks indicate statistical significance (*0.01 < P < 0.05, one-way ANOVA). (**c**) Symptoms of the *VqTLP29* transgenic lines and Col-0 3 days post *B*. *cinerea* inoculation were scored by defining three lesion classes (<40%, 40–80%, >80%). The black part represents percentage of *B*. *cinerea* induced necrotic lesion sizes less than 40%, the white part represents percentage of lesion sizes between 40% to 80%, and the gray part represents percentage of lesion sizes more than 80%. The sum of the percentages in three parts is 100%. (**d**) Cell death, H_2_O_2_ and O^2−^ accumulation in the *VqTLP29* transgenic lines and Col-0 72 h post *B*. *cinerea* inoculation. Three infected leaves of the transgenic lines and Col-0 were required for each stain in three independent experiments. (**e**) Expression levels of disease resistance genes in the *VqTLP29* transgenic lines and Col-0 at 0, 12, 24 and 48 hpi following *B*. *cinerea* inoculation. Asterisks indicate statistical significance (*0.01 < P < 0.05, **P < 0.01, one-way ANOVA). The experiments were repeated three times with consistent results.

Detached leaves from the *VqTLP29* over-expressing line L1, L2 and L3 inoculated with *B*. *cinerea* had more disease symptoms than those from Col-0 (Fig. 5a). Three days after inoculation, *B*. *cinerea* induced necrotic lesions were evident on the entire leaf of transgenic lines and were larger than those found on Col-0 (Fig. 5b). Symptoms were scored by defining three lesion classes (<40%, 40–80%, >80%). Percentages of lesion sizes over 40% in white and grey parts were shown in the transgenic line L1, L2 and L3 with 92%, 88% and 86%, respectively, while minimum 44% of lesion sizes over 40% were observed in Col-0 (Fig. 5c). A histochemical staining assay also showed that the extent of cell death, and levels of H_2_O_2_ and O^2−^ were higher in the three transgenic lines than in Col-0 (Fig. 5d). The expression levels of *VqTLP29* following *B*. *cinerea* inoculation decreased in the three transgenic lines (Supplementary Figure S2c), while the expression levels of *PR1* and *NPR1* were on peak at 48 hpi, and the expression of *ICS1* began to increase gradually after the lowest value at 12 hpi. The expression levels of *PR1*, *NPR1* and *ICS1* in the transgenic lines were consistently higher than in Col-0. The expression levels of *PDF1*.*2*, a downstream gene in the JA/ET signaling pathway^35^, gradually increased in the transgenic lines following infection, with the highest value measured at 48 hpi, and the expression levels of *LOX3* decreased slightly during the onset of disease, but always remained higher than in Col-0 (Fig. 5e).

### Response of *VqTLP29* over-expressing *A*. *thaliana* lines to *Pst* DC3000 inoculation

**Figure 6:**
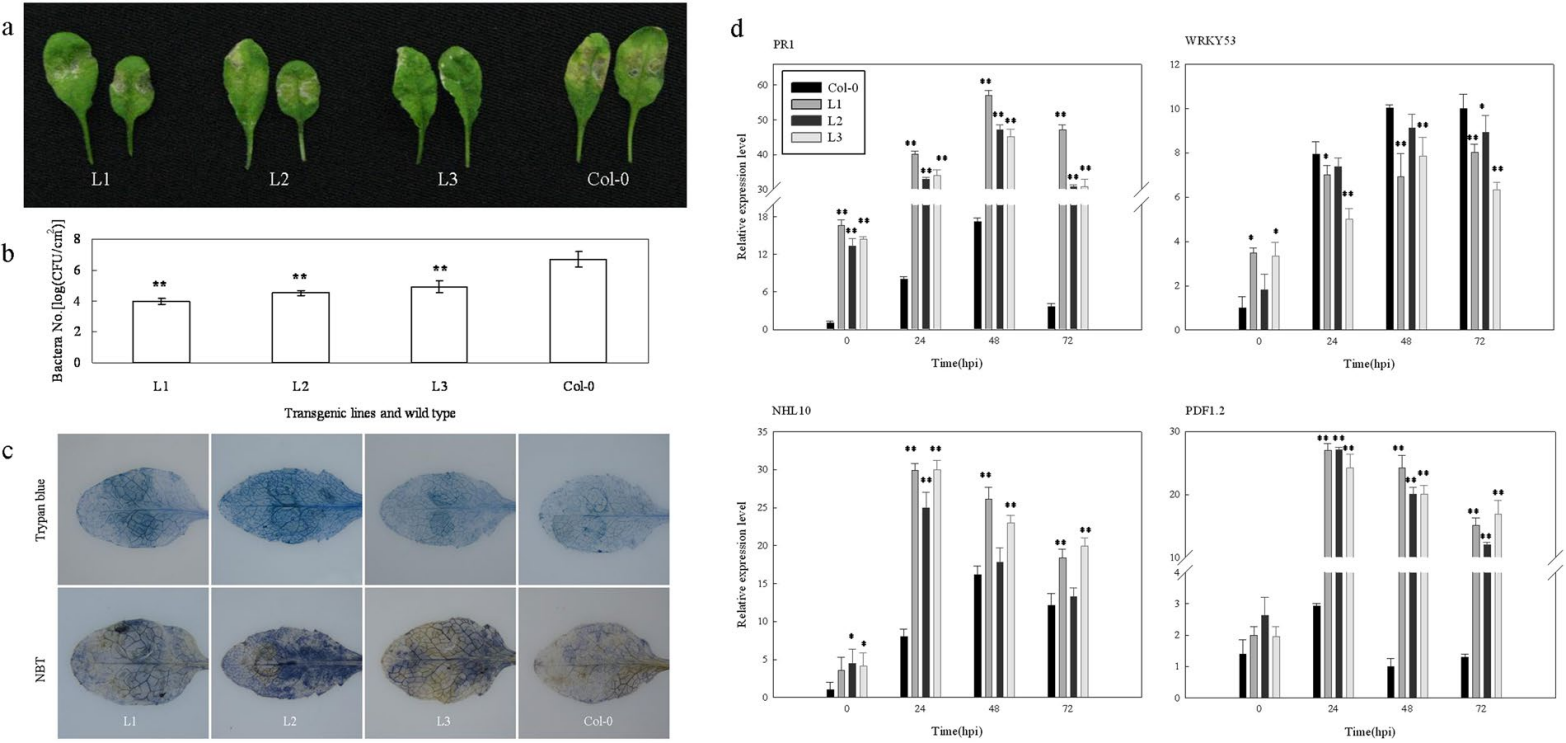
Response of *VqTLP29* over-expressing *Arabidopsis thaliana* lines to *Pst* DC3000 inoculation. (**a**) Disease symptoms in representative rosettes of the 4-week old *VqTLP29* transgenic line L1, L2, L3 and Col-0 after DC3000 inoculation for 3 days. Leaves were injected with the DC3000 suspension using 1 mL needless syringes and kept highly moisturizing until the lesion shown. (**b**) The number of bacteria of the *VqTLP29* transgenic lines and Col-0 3 days post DC3000 inoculation. The colony counting assay of the transgenic lines and Col-0 was carried out in three independent experiments. Asterisks indicate statistical significance (**P < 0.01, one-way ANOVA). (**c**) Cell death and O^2−^ accumulation in the *VqTLP29* transgenic lines and Col-0 72 h post DC3000 inoculation. Three infected leaves of the transgenic lines and Col-0 were required for each stain in three independent experiments. (**d**) Expression levels of disease resistance genes in the *VqTLP29* transgenic lines and Col-0 at 0, 24, 48 and 72 hpi following DC3000 inoculation. Asterisks indicate statistical significance (*0.01 < P < 0.05, **P < 0.01, one-way ANOVA). The experiments were repeated three times with consistent results.

To elucidate the role of *VqTLP29* in bacterial resistance, the three *VqTLP29* over-expressing line L1, L2 and L3 were inoculated with the bacterium *Pseudomonas syringae* pv. DC3000. Three days after inoculation, disease symptoms were less apparent in the transgenic lines than in Col-0 (Fig. 6a), which exhibited yellow spots at 24 hpi and dry leaves were curled and brittle after 3 days. Moreover, the quantities of bacteria were lower in the three transgenic lines than in Col-0 (Fig. 6b), while the frequency of cell death and the degree of O^2−^ accumulation were both higher (Fig. 6c). The expression levels of *VqTLP29* following DC3000 inoculation increased in the three transgenic lines (Supplementary Figure S2b), and *PR1* expression was higher in the transgenic lines than in Col-0, peaking at 48 hpi. The transcriptional regulators *WRKY53* ^36^ and *NHL10* (NDR1/HIN1-like 10)^37^ are known to play roles in the *A*. *thaliana* SA pathway. The expression levels of *WRKY53* were lower in the transgenic lines from 24–72 hpi than in Col-0, and the expression levels of *NHL10* were higher in the transgenic lines, peaking at 24 hpi. The expression levels of *PDF1*.*2* involved in the JA/ET signaling pathway were higher in the transgenic lines than in Col-0, with a peak at 24 hpi (Fig. 6d).

### Stomatal closure immunity response

**Figure 7:**
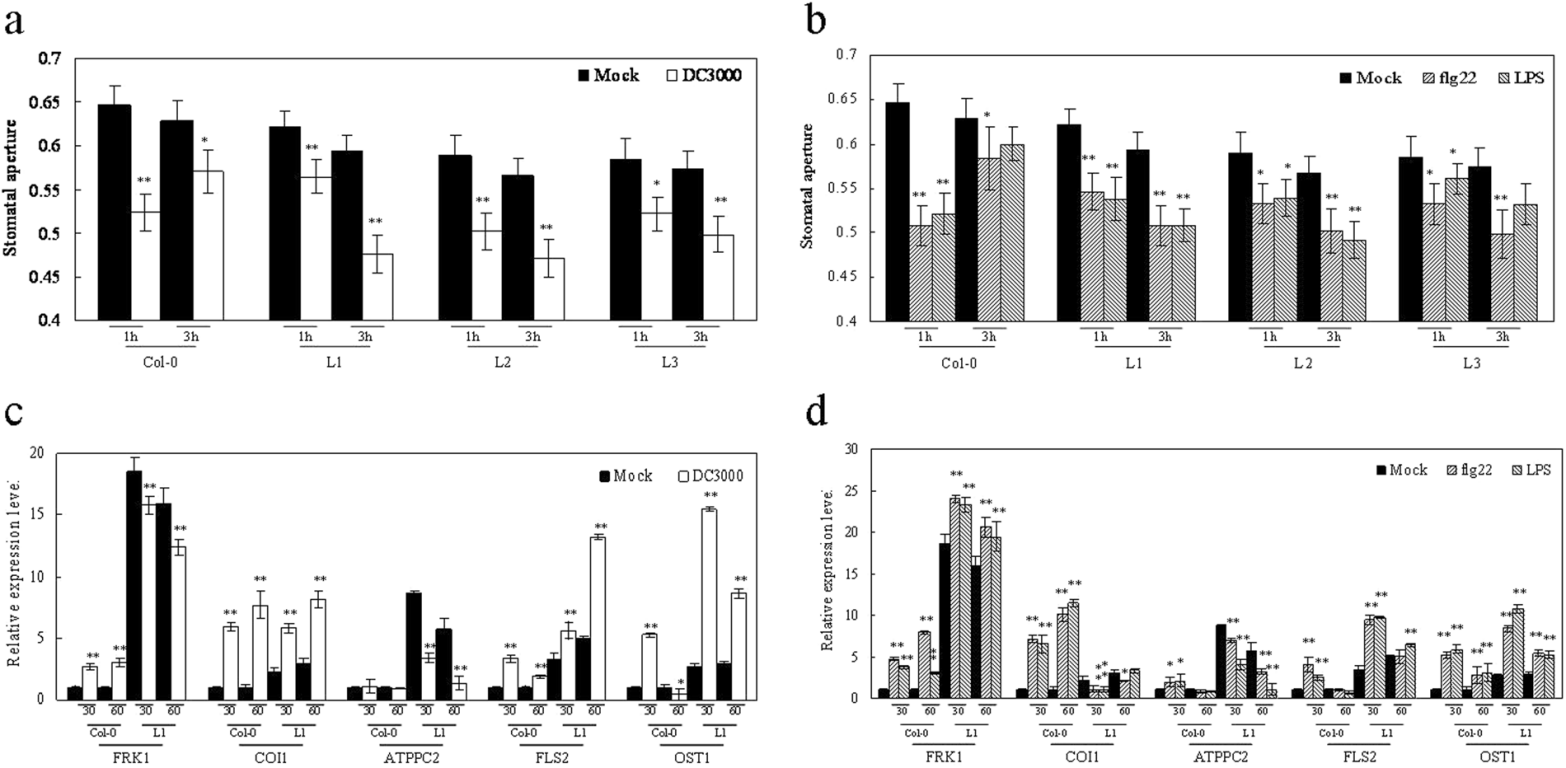
Altered stomatal immunity response in *VqTLP29* over-expressing *Arabidopsis thaliana* lines. (**a**,**b**) Stomatal apertures (width/length; µm) in epidermal peels of the leaves in the 4-week old *VqTLP29* transgenic line L1, L2, L3 and Col-0 after 1 h and 3 h of incubation with (**a**) Mock sample (MgCl_2_) and DC3000; (**b**) Mock (MgCl_2_), flg22 and LPS. Data represent mean values ± SD with 60 stomata per sample. Stomatal apertures of the transgenic lines and Col-0 were combined and analyzed with the microscope and software Image-J in three independent experiments. Data represent mean values ± SD with 60 stomatal per sample. Asterisks indicate statistical significance (*0.01 < P < 0.05, **P < 0.01, one-way ANOVA). (**c**,**d**) Expression levels of stomatal immunity response related genes in the *VqTLP29* transgenic line L1 and Col-0 after 30 min and 60 min of incubation. Asterisks indicate statistical significance (*0.01 < P < 0.05, **P < 0.01, one-way ANOVA). The experiments were repeated three times with consistent results.

Stomatal closure is known to be part of the induced plant innate immunity response and serves to limit pathogen infection^38^. In this study, we observed a marked reduction in leaf stomatal aperture in both the *VqTLP29* over-expressing *A*. *thaliana* line L1, L2 and L3 and Col-0 during the first hour after DC3000 inoculation. However, after 2 hours of continued incubation, stomata reopened in Col-0, but not in the transgenic lines (Fig. 7a). It has been shown that the bacterium derived molecules, flg22 and LPS, act as pathogen-associated molecular patterns (PAMPs) to stimulate/induce the innate immunity in plants^39, 40^. We observed that within the first hour of incubation with flg22 or LPS, marked reduction in stomatal aperture were observed in all transgenic lines and Col-0. During the subsequent hours, the stomata in Col-0 reopened, while stomatal apertures still decreased in the transgenic lines (Fig. 7b).

Next we measured the expression of genes known to be involved in modulating stomatal guard cell movement or associated with signal transduction pathways in *VqTLP29* over-expressing line L1 (Fig. 7c,d). *FRK1* is flg22-induced receptor kinase involved in the SA pathway, and *COI1* can activate the JA signaling pathway, and is used as an inhibitor of stomatal closure^41^. *ATPPC2* acts directly upon stomatal closure in the abscisic acid (ABA) pathway^42^. *FLS2* is a flagellin receptor. *OST1* is a guard-cell-specific kinase. *FLS2* and *OST1* are required for perception of bacterial surface molecules in *A*. *thaliana* stomatal guard cells^38^. The expression levels of *FRK1* were higher in L1 than in Col-0 under normal condition. *FRK1* expressions decreased in L1 from 30–60 min post DC3000 inoculation and remained higher than in Col-0. The expression levels of *COI1* increased in both L1 and Col-0 following DC3000 inoculation. The expression levels of *ATPPC2* were higher in L1 than in Col-0 under normal condition. *ATPPC2* expression decreased in L1 after infection but still higher than in Col-0. The expression levels of *FLS2* and *OST1* increased in L1 after DC3000 inoculation, with *FLS2* attained its peak at 60 min and *OST1* attained its peak at 30 min. After treated with flg22 or LPS, the expression levels of *FRK1* increased in L1 and Col-0. The expression levels of *COI1* decreased in L1 after inoculation but increased in Col-0. The expression levels of *ATPPC2* decreased gradually in L1 after inoculation and still higher than in Col-0. The expression levels of *FLS2* increased in L1 peaking at the 30 min after inoculation. And the expression of *OST1* also increased in L1 and Col-0, with the maximum values at 30 min after flg22 or LPS inoculation.

**Figure 8:**
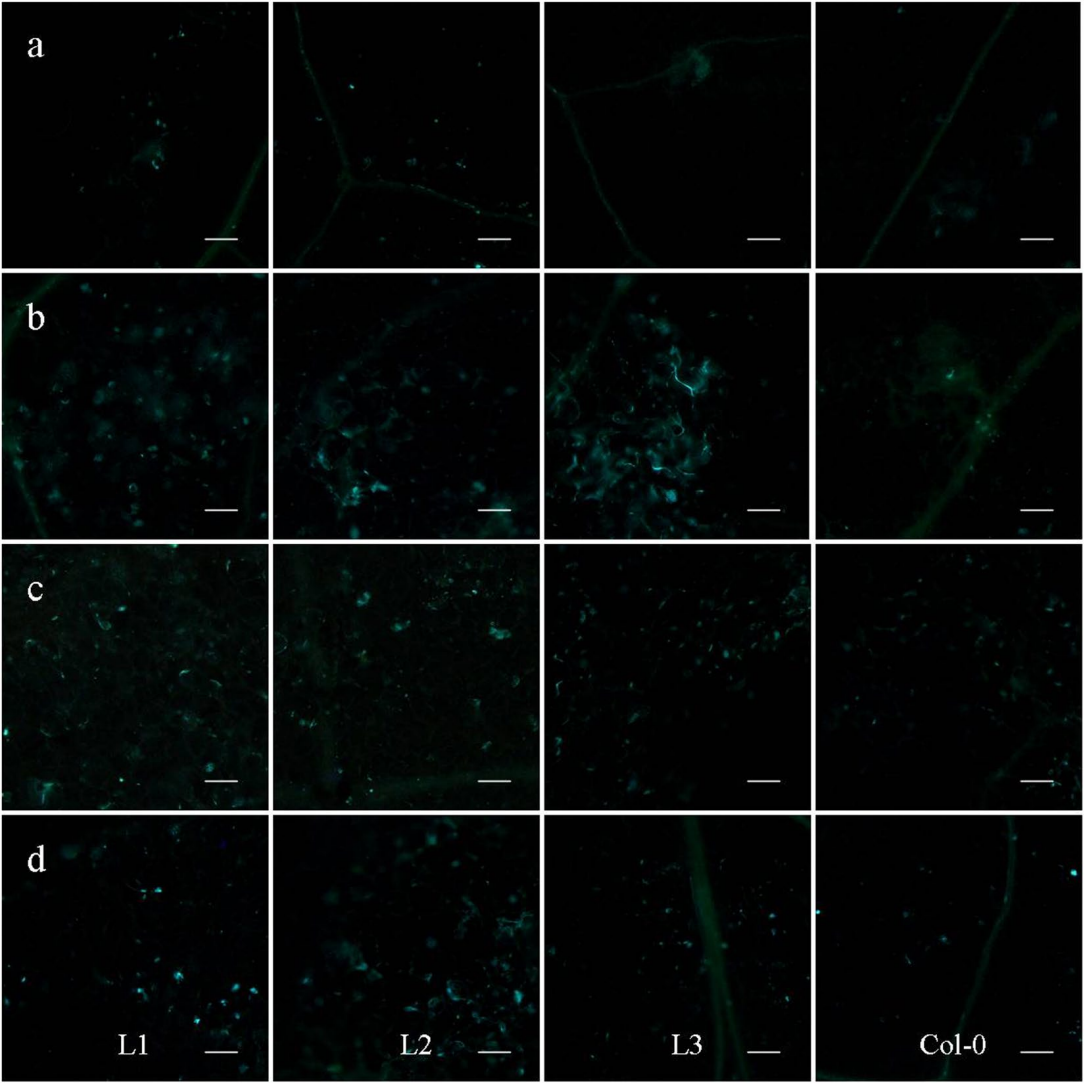
Callose deposition in *VqTLP29* over-expressing *Arabidopsis thaliana* leaves. (**a**–**d**) Leaves of the 4-week old *VqTLP29* transgenic line L1, L2, L3 and Col-0 were stained with aniline blue after infiltration for 18 h with (**a**) Mock sample (MgSO_4_), (**b**) DC3000, (**c**) flg22 and (**d**) LPS, and photographed with the microscope. Three diseased leaves of the transgenic lines and Col-0 were required in three independent experiments. The scale bar in the figure indicates 100 μm.

Staining the leaves of different genotypes with aniline blue indicated that more callose were produced in response to DC3000 inoculation, and treatments with flg22 or LPS in the transgenic line L1, L2 and L3 than in Col-0 (Fig. 8).

## Discussion

The *TLP* family has been extensively studied in animals and fungi^43^ and some TLP genes in plants are known to be involved in defense against pathogens^10–12, 17^. In the current study, 33 grape *VvTLP* genes were identified (Table 1) and analyzed into four subfamilies through the phylogenetic analysis (Fig. 1). The thaumatin domain was present in 27 of the 33 grape *TLP* genes from Type I and II subfamily, while was not in 6 genes (*VvTLP1*, *VvTLP2*, *VvTLP14*, *VvTLP17*, *VvTLP25* and *VvTLP27*) from Type III and IV subfamily. Four of the 33 *VvTLP* genes (*VvTLP4*, *VvTLP5*, *VvTLP14* and *VvTLP21*) did not have accession numbers in support of the expressed sequence tag (EST) data. However, structural analyses indicated that they contain the conserved thaumatin domain coding sequence, with the exception of *VvTLP14*, and they were included into the *TLP* family^44^. Gene duplication events play a major role in grape genome rearrangement and expansion (Fig. 2)^45^, and segregation duplication events have been shown to provide a reference for the evolutionary relationship between *TLP* genes, thereby enabling functional predictions^46^. To verification the roles of grape *TLP* genes in signaling pathways related to pathogen induced stress^22^, we evaluated their expression in grape cultivars that had been infected with the biotrophic mycoparasite *E*. *necator*, the hemi-biotrophic mycoparasite anthracnose and *B*. *cinerea*, which has saprophytic growth (Fig. 3). The expression of cisgenic *VVTL1* proved as a secerted protein in grape was shown to significantly inhibit the growth of hyphae of both *E*. *ampelina* and *E*. *necator* ^18, 19^, which was supported in this study with the increased expression after pathogens treatments. Other *TLP* genes have also been shown to increase resistance to plant diseases^10–12^, indicating a substantial role for the *TLP* genes in the regulatory networks involved in pathogen infection. The analysis of gene expression profiles often provides useful clues for functional assessment. The homolog of *TLP29* from the wild grape species *V*. *quinquangularis* ‘Shang-24’ was aimed for cloning and for additional functional studies. Analysis of *VqTLP29* protein sequence showed that *VqTLP29* protein was a secerted protein, and its signal peptide cleavage site was located between 23rd and 24th amino acid. Although the protein sequence from 172nd to 204th amino acid were defined as the nuclear localization signal assessed as three points with cNLS on line shown in Supplementary Data S2, the subcellular localization experiment was analyzed and confirmed that *VqTLP29* protein was located in the cytoplasm in grape mesophyll protoplasts shown in Supplementary Figure S4 with percentages of the protoplast transfection shown in Supplementary Table S3. Besides, over-expression of *VqTLP29* in *Arabidopsis thaliana* enhanced the resistance to powdery mildew and the bacterium *Pseudomonas syringae* pv. tomato DC3000, but decreased resistance to *B*. *cinerea*.

The antifungal protein *VqTLP29* encoded the thaumatin-like protein similar with PR5 protein mainly acted on the late stage of defensive reaction. Over-expression of PRs would reduce only a limited number of diseases, depending on the nature of the protein, plant species, and pathogen involved^47^. Over-expression of PR5 could enhance resistance to biotic and abiotic response with activating many defense genes in SA or JA/ET signaling pathway. The genotypes of *VqTLP29* were shown higher expression values in transgenic lines under normal growth with the increased expression levels of SA-defense genes (*PR1*, *NPR1* in Figs 4d, 5e and 6d) and SA-synthesis gene (*ICS1* in Figs 4d and 5e). Higher expression levels of *PR1* were reported and shown in over-expression of transgenic factor Di19 or *CPK11* (Ca^2+^-dependent protein kinase 11) plants under normal condition, respectively^48^. Higher expression levels of *NPR1* were also shown in over-expression of *GhMKK5* plants under normal conditions, which belonged to MAPK kinase (MAPKKs)^49^. And the transcripts of *ICS1* were repressed 8-fold in *GmMPK4*-silenced plants with *GmMPK4* negatively controlling SA^33^. All above results were similar in this study and the data proved that over-expression of *VqTLP29* had already activated SA signaling pathway under normal condition. The genotypes of *VqTLP29* were shown higher expression values in transgenic lines under normal condition with the expression levels of JA-synthesis gene (*LOX3* in Figs 4d and 5e) being enhanced over 10-fold, while that of JA/ET-defense downstream gene (*PDF1*.*2* in Figs 5e and 6d,c) had no significant change. Activated *NPR1* could ultimately lead to the activation of some SA-responsive genes but would act as a cytosolic function of inhibition of JA signaling pathway, which lead to lose the ability of activating JA-responsive genes^32^. Over-expression of *VqTLP29* with higher *NPR1* could not activate JA-responsive genes like *PDF1*.*2* as referred by these studies^35, 50^. It is evident that *LOX3* was a component in JA synthesis and used as an important lipoxygenase in JA synthesis^34, 51^. Although the expression level of *LOX3* was over 10-fold higher in 35S:MYC3 transgenic lines than wild type under normal condition, the expression of *PDF1*.*2* was not changed^52^. These data proved that over-expression of *VqTLP29* had already activated JA synthesis while had no effect on JA signaling pathway in plants under normal growth.

We assessed the *VqTLP29* transgenic lines with responses to two fungal pathogens and a bacterium. The phenotypes of transgenic and wild type plants were similar. *VqTLP29* transgenic lines clearly improved resistance to powdery mildew and DC3000 with increased *VqTLP29* expression, but increased susceptibility to *B*. *cinerea* with decreased *VqTLP29* expression. The phytohormone SA plays an important role in limiting the invasion of powdery mildew and *B*. *cinerea* ^53–55^. Studies showed that the expression level of *PR5* increased post powdery mildew inoculation with increased expressions of *PR1* and *NPR1* ^21, 56–58^. We observed that the expressions of *PR1* and *NPR1* increased significantly in the transgenic lines following the three pathogens inoculation, which suggested that over-expression of *VqTLP29* caused an up-regulation of the SA signaling pathway. The expression level of *LOX3* gradually decreased to the lowest level at 120 hpi post powdery mildew inoculation but remained higher in the transgenic lines, indicating that over-expression of *VqTLP29* could promote JA biosynthesis following powdery mildew inoculation (Fig. 4d). The immune response against *B*. *cinerea* is also known to be regulated by JA/ET signaling, suggesting that the ethylene and JA pathways interact with each other, co-regulating the expression of defense related genes^59, 60^. We observed that the expression of *PDF1*.*2* increased after *B*. *cinerea* inoculation, revealing that the JA/ET pathway was induced in the transgenic lines (Fig. 5e), which is supported by two studies with the same result of *PDF1*.*2* expression^61, 62^. The expression level of *LOX3* was higher in the transgenic lines, indicating that over-expression of *VqTLP29* could promote JA biosynthesis following *B*. *cinerea* inoculation (Fig. 4d). *ICS1* expression appears to decrease at 12–24 hpi post powdery mildew and *B*. *cinerea* inoculation, which showed that *ICS1* was cycling in response to the over-expression of *VqTLP29* ^33, 56, 57^. *WRKY53*, a member of the WRKY transcription factor family, has been shown to be involved in pathogen-triggered SA signaling^36^, as has *NHL10* ^37^. We noted an up-regulation of both genes after DC3000 inoculation in parallel with increased expression of *PDF1*.*2*, but the expression levels of *WRKY53* were lower in the transgenic lines than in Col-0 (Fig. 6), which implied that over-expression of *VqTLP29* has effects on SA or JA/ET pathway with DC3000 inoculation. Taken together, the expression data suggest that over-expression of *VqTLP29* acts as a regulator that differentially modulates immunity against powdery mildew, *B*. *cinerea* and DC3000 via the SA or JA/ET signaling pathway.

Stomatal defense against bacterial invasion is an important component of the innate immunity, and is a target of virulence factors produced by DC3000. Here we used several marker genes involved in this process to investigate the role of *VqTLP29* in bacterial resistance. *FRK1* transcript levels were higher in the transgenic line L1 than Col-0 (Fig. 7c,d), which is consistent with the innate immune response in *A*. *thaliana* leaves being activated via an SA-dependent mechanism^63^. The expression levels of both *FLS2* and *OST1* increased in L1 after different treatments, while *ATPPC2* expression decreased. *FLS2* has been shown to activate resistance to DC3000^39^, and involved in ABA signaling pathway to act directly on guard cells inducing stomatal closure by promoting the efflux of potassium and anions and the removal of organic osmolytes^42^. The mechanism by which *VqTLP29* regulates stomatal response to PAMPs can be explored with the characterization that how it mediates gene regulation. Our studies showed that the stomatal response in *VqTLP29* over-expressing *A*. *thaliana* was up-regulated by the SA signaling pathway and down-regulated by ABA signaling pathway.

In conclusion, TLP gene expression is broadly influenced by *E*. *ampelina*, *E*. *necator* and *B*. *cinerea* inoculations, indicating the existence of a complex regulatory network that responds to biotic stress. In addition, we identified the role of *VqTLP29* from disease resistant grape *V*. *quinquangularis* cv. ‘Shang-24’ in responses to various pathogens. Over-expression of *VqTLP29* in *A*. *thaliana* had already activated SA signaling pathway, JA synthesis and had no effect on JA signaling pathway under normal condition. *VqTLP29* over-expressing lines enhanced resistance to the powdery mildew and DC3000 but increased susceptibility to *B*. *cinerea*, with up-regulating the SA and JA/ET signaling. Data from these analyses will be useful in defining the transcriptional networks that are regulated by *VqTLP29* during immune responses against pathogens.

## Methods

### Plant materials

In this study, grape genotype ‘Shang-24’ (*V*. *quinquangularis*), ‘Hunan-1’ (*V*. *pseudoreticulata*), ‘Shuangyou’ (*V*. *amurensis*) and Red Globe (*V*. *vinifera*) were used for analysis of disease resistance in the grape germplasm resources orchard of Northwest A&F University, Yangling, Shaanxi, China. Samples of grape organs were obtained from ‘Shang-24’. *A*. *thaliana* (transgenic lines and wild type Col-0) plants were grown at 21–22 °C, and 70% relative humidity under long day (8 h dark, 16 h light) conditions. For all experiments, 4-week old plants were used. All experiments were repeated in triplicate and all samples were immediately frozen in liquid nitrogen and stored at −80 °C until further use.

### Identification and annotation of grape *TLP* genes

A profile of the TLP DNA-binding domain (PF00314) was downloaded from the Pfam protein family database (http://pfam.sanger.ac.uk/)^64^ and used to identify putative *TLP* genes from the grape genome sequence (http://www.genoscope.cns.f)^65^. The deduced *TLP* genes were annotated based on their respective chromosome distribution^66^ and their sequences were confirmed using an in-house transcriptome database.

### Bioinformatic analysis of grape *TLP* genes

A phylogenetic tree of the 33 predicted *VvTLP* genes was constructed with MEGA5 software using the neighbor-joining method^67^, and their exon-intron structures were determined based on alignments of the coding regions and full-length sequences (http://www.genoscope.cns.fr/externe/GenomeBrowser/Vitis/). Diagrams of the gene structures were generated using the Gene Structure Display Server 2.0 (http://gsds.cbi.pku.edu.ch), and protein structures using PROSITE (http://prosite.expasy.org/). *TLP* gene duplications were identified as previously described^66^, and syntenic blocks were used to construct a synteny analysis map of the *VvTLP* genes from the Plant Genome Duplication Database^68^. Diagrams were generated using Circos version 0.63 (http://circos.ca/). The results of semi-quantitative RT-PCR were analyzed and quantified using the Gene Tools software, and the relative expression levels of *VvTLP* genes under different treatments compared to the controls were used for hierarchical cluster analysis with MeV 4.8.1^69^.

### Grape disease assays

The anthracnose (*E*. *ampelina*) was isolated and sporulated on potato dextrose agar (PDA) at 25 °C. Spores were suspended in sterile water and 0.5 ml of the suspension (2.0 × 10^6^ spores ml^−1^) was sprayed onto each side of the young leaves from three vines of ‘Shang-24’ and Red Globe. Sterile water was used as a control at the same time points. Samples were collected at 0, 6, 12, 24, 48, 72 and 120 hours post inoculation (hpi)^30^. Powdery mildew (*E*. *necator*) was used to inoculate young leaves of ‘Shang-24’ and ‘Hunan-1’, and sterile water was used as a control at the same time points. Samples were collected at 0, 6, 12, 24, 48, 72, 96, and 120 hpi^16^. *B*. *cinerea* was isolated from susceptible leaves in the field and maintained on potato glucose agar medium in the dark for 3 weeks at 23 °C. Conidia were washed with sterile water and a concentration of 2.0 × 10^6^ spores ml^−1^ was used for the experiment. Sterile water was used as a control at the same time points. Leaves were sampled at 4, 8, 12, 18, 24, 36, 48, 72 and 96 hpi^31^.

### *A*. *thaliana* disease assays

*A*. *thaliana* powdery mildew (*Golovinomyces cichoracearum* UCSC1) was preserved by growing it in the *phytoalexin deficient* 4 mutant^40^. Sterile water was sprayed on the surface of the *VqTLP29* transgenic line L1, L2, L3 and Col-0 before inoculations using the leaf pressing method^70^. Samples were collected at 0, 24, 48, 72, 96, 120, 144, and 168 hpi. A spore counting assay was carried out 7 days post powdery mildew inoculation^71^. Spore suspensions were extracted and diluted in sterile water with 10 infected leaves and then counted using a hemocytometer under the microscope. *B*. *cinerea* was sporulated on PDA at 25 °C for 3 weeks and then spores were suspended in sterile water with 4% maltose and 1% peptone^72^ to a concentration of 2.0 × 10^6^ spores ml^−1^. Leaves were detached and inoculated by dropping 10 μl of spore suspension onto the adaxial surface^73^. Samples were collected at 0, 12, 24, 48 and 72 hpi, and the percentage of lesion areas with respect to the whole leaf was determined using grid statistics 3 days after infection. DC3000 was cultured overnight at 28 °C in LB medium with 1/2 salt concentration (pH 7.0, 10 g L^−1^ tryptone, 5 g L^−1^ yeast extract powder and 5 g L^−1^ NaCl). Bacterial cultures with an OD_600_ of 0.6 were collected by centrifugation (12,000 g, 10 min) and resuspended in 10 mM MgCl_2_ containing 0.005% Silwet L-77 (OSI Specialties, Sigma) to a final OD of 0.02. Leaves were injected with the DC3000 suspension using 1 mL needless syringes^74^, and samples were collected at 0, 24, 48, 72 and 96 hpi. A colony counting assay was carried out 3 days post DC3000 inoculation^75^.

Histochemical staining assay was conducted with leaves 7 days post powdery mildew infection, 3 days post DC3000 inoculation and 3 days post *B*. *cinerea* inoculation^76^. Nine susceptible leaves from the *VqTLP29* transgenic lines and Col-0 were collected to visualize callose deposition by staining with 1% (w/v) aniline blue dissolved in 150 mM K_2_HPO_4_ (pH 9.5); cell death with trypan blue; O^2−^ accumulation with 6 mM nitro blue tetrazolium (NBT); and H_2_O_2_ with 1 mg ml^−1^ diaminobenzidine (DAB, pH 3.8). Infected leaves were first bleached overnight in 95% ethanol until transparent and then transferred to the aniline blue solution for 24 h prior to microscopic (Olympus BX53, Tokyo, Japan) observation under UV illumination. The trypan blue buffer consisted of 20 ml ethanol, 10 ml phenol, 10 ml ddH_2_O, 10 ml lactic acid and 10 mg trypan blue. Infected leaves were boiled in trypan blue solution for 5 min and then bleached in 2.5 mg ml^−1^ chloral hydrate for 48 h. NBT was dissolved in HEPES buffer (pH 7.5). Infected leaves were soaked in NBT solution for 2 h and DAB for 8 h, and then transferred into 80% ethanol at 60 °C for 2 h and finally held at room temperature for 48 h. Disease related genes used to assess the response to these treatments and the corresponding gene specific primers used for semi-quantitative PCR primers are listed in Supplementary Table S2.

### Response of stomata to different treatments

To ensure that 80% of the stomata were open at the onset of the experiments, *A*. *thaliana* plants were placed in the light (100 μmol m^−2^ s^−1^) for 3 h. The epidermis of 3 fully expanded young leaves from the *VqTLP29* transgenic lines and Col-0 was peeled off manually and immediately immersed in 10 mM MgCl_2_ (mock treatment), DC3000 suspension (OD_600_ of 0.02 in MgCl_2_), 5 μM flg22 (Flagellin Fragment, peak area by HPLC ≥95%, Anaspec, USA) in MES buffer (25 mM MES-KOH, pH 6.15 and 10 mM KCl) and 100 ng μl^−1^ LPS (lipopolysaccharide, Sigma-Aldrich, dissolved in MgCl_2_)^40, 75^. At 1 h and 3 h time points, treated epidermal peel samples were observed under a microscope (Olympus BX53). Stomatal transverse length and longitudinal width were measured using Image-J. Four treatments were conducted for the transgenic lines and Col-0 using 1 mL needle syringes. Treated leaves were sampled separately at 30 and 60 min and frozen in liquid nitrogen. Leaves of the transgenic lines and Col-0 18 h post treatments were stained with aniline blue to detect callose deposition.

### Vector construction

Total ‘Shang-24’ RNA and cDNA was isolated as described below. The *VqTLP29* coding sequence fragment (735 bp, Supplementary Data S1) was amplified by PCR using the primers VqTLP29-F (5-GCTCTAGAATGGGGATGCTGCT-3) and VqTLP29-R (5′-GGGGTACCCTAGTGGTGAGGG-3) with *Xba*I and *Kpn*I sites (underlined) included. The PCR reactions were carried out as follows: 94 °C for 5 min; 35 cycles at 94 °C for 30 s, 58 °C for 30 s, 72 °C for 50 s; 72 °C for 2 min, and the PCR product was cloned into the pGEM-T Easy vector (Promega, Madison, WI, USA). The resulting plasmids were sequenced by Sunny Biotechnology Co. Ltd (Shanghai, China). The *VqTLP29* coding sequence PCR product and the pCAMBIA2300-35S vector stored in our lab were both digested with the *Xba*I/*Kpn*I restriction endonucleases and co-ligated overnight using the DNA Ligation Kit Ver2.1 (Tiangen company, Beijing, China).

### Generation of transgenic *A*. *thaliana* plants over-expressing *VqTLP29*

The binary plasmid pCAMBIA2300-35S-*VqTLP29* was transformed into *Agrobacterium tumefaciens* (strain GV3101) stored in our lab. The transformed cells were pelleted by centrifugation (4,000 g, 10 min) and resuspended in a 5% (w/v) sucrose solution containing 0.05% Silwet L-77, to a final OD_600_ of 0.6 and used to transform *A*. *thaliana* plants by the floral dip method^77^. Screening of kanamycin-resistant transgenic *A*. *thaliana* seedlings from the T1 generation and confirmation of the presence of the *VqTLP29* transgene in three transgenic lines is described in Supplementary Figure S3.

### RNA isolation, sqRT-PCR and qRT-PCR

Total RNA was extracted using an EZNA Plant RNA Kit (R6827-01, Omega Bio-tek, USA). First-strand cDNAs were synthesized using a PrimeScript 1st Strand cDNA Synthesis Kit (TaKaRa Biotechnology, Dalian, China) and diluted 12-fold. *VvActin* and *EF1-α* (GenBank accession number AY680701 and EC931777, respectively) were used for the semi-quantitative RT-PCR analysis with the primers F1 (5′-GATTCTGGTGATGGTGTGAGT-3′), R1 (5′-GACAATTTCCCGTTCAGCAGT-3′) and F2 (5′-AGGAGGCAGCCAACTTCACC-3′), R2 (5′-CAAACCCTGCATCACCATTC-3′). Gene-specific primers for each *VvTLP* gene were designed using Primer Premier 5.0 and optimized using oligo 7 (Supplementary Table S1). PCR reactions were as follows: 94 °C for 5 min; 28–40 cycles at 92 °C for 30 s, 58 ± 4 °C for 30 s, 72 °C for 30 s; 72 °C for 2 min. qRT-PCR was conducted using SYBR Green (TaKaRa Biotechnology) with a CFX Connect Real-Time PCR instrument (Bio-Rad, Hercules, CA, USA) with a final volume of 20 μl per reaction. Each reaction mixture consisted of 10.0 μl SYBR Premix Ex Taq II, 1.0 μl cDNA, 0.8 μl each primer (10 μM), and 7.4 μl sterile H_2_O. Cycling parameters were: 95 °C for 30 s; 40 cycles at 95 °C for 5 s, 60 °C for 30 s. Melt-curve analyses was performed using a program with 95 °C for 15 s and then a constant increase from 60 °C to 95 °C. The software CFX_Manager was used to analyze the relative expression levels with significance analysis of 2−ΔΔCt.

### Statistical analysis

Data are presented as means and standard errors using Microsoft Excel and SigmaPlot 10.0. One-way ANOVA analysis was performed using the SPSS Statistics 17.0 software (IBM China Company Ltd. Beijing, China) to assess significant differences.

## Electronic supplementary material

Supplementary Information (2017-05)
