# Supplementary material for: Analysis of the grape (Vitis vinifera L.) thaumatin-like protein (TLP) gene family and demonstration that TLP29 contributes to disease resistance
Source: Sci Rep. 2017 Jun 27;7:4269. doi: 10.1038/s41598-017-04105-w (PMC5487326; doi:10.1038/s41598-017-04105-w)
Supplement: Supplementary file 1 — Supplementary Information (2017-05) [file 41598_2017_4105_MOESM1_ESM.pdf]

Supplementary Information

Analysis of the grape (*Vitis vinifera* L.) thaumatin-like protein (TLP) gene family and demonstration that *TLP29* contributes to disease resistance

Xiaoxiao Yan[1, 2], Hengbo Qiao[1, 2], Xiuming Zhang[1, 2], Chunlei Guo[1, 2], Mengnan Wang[1, 2], Yuejin Wang[1, 2], Xiping Wang[1, 2*]

[1]State Key Laboratory of Crop Stress Biology in Arid Areas, College of Horticulture, Northwest A&F University, Yangling, Shaanxi 712100, China

[2]Key Laboratory of Horticultural Plant Biology and Germplasm Innovation in Northwest China, Ministry of Agriculture, Northwest A&F University, Yangling, Shaanxi 712100, China

*Corresponding author: Xiping Wang

College of Horticulture, Northwest A & F University,

Yangling 712100, Shaanxi, China

E-mail: wangxiping@nwsuaf.edu.cn

Tel: 86-29-87082429

Fax: 86-29-87082613

Supplementary Methods

Grape disease assays of samples collection

After anthracnose (*Elsinoe ampelina*) inoculation on the leaves of grape 'Shang-24' and Red Globe with sterile water as the control treatment, nine leaves from three separate vines were consisted of one sample at each time point[15, 30].
After powdery mildew (*Erysiphe necator*) inoculation on the leaves of grape 'Shang-24' and 'Hunan-1' with sterile water as the control treatment, nine leaves from three separate vines were consisted of one sample at each time point[16].
Conidia of *B. cinerea* were pregerminated for 2 h at 23°C before inoculations were performed with grape 'Shuangyou' and Red Globe. Sterile water was sprayed as control at the same time. Both the infected and control leaves were sampled 0, 4, 8, 12, 18, 24, 36, 48, 72 and 96 hpi[31].
Biological replicates experiments of above three pathogens treatments were performed in triplicate and all samples above for semi-quantitative RT-PCR and real-time quantitative PCR were immediately frozen in liquid nitrogen and stored at -80℃.

Vector construction of subcellular localization

Total 'Shang-24' RNA and cDNA was isolated as described below. The *VqTLP29* coding sequence fragment was amplified by PCR using the primers VqTLP29-F1 (5-GC<u>TCTAGA</u>ATGGGGATGCTGCT-3) and VqTLP29-R1 (5′-GG<u>GGTACC</u>GTGGTGAGGG-3) with *Xba*I and *Kpn*I sites (underlined) included. The PCR reactions were same as the description before. The methods of the PCR product being sequenced were also same as the description before. This *VqTLP29* coding sequence PCR product and the PBI221-GFP vector stored in our lab were both digested with the *Xba*I/*Kpn*I restriction endonucleases and co-ligated overnight using the DNA Ligation Kit Ver2.1 (Tiangen company, Beijing, China).

Analysis of *VqTLP29* protein sequence

The signal peptide and transmembrane helices in *VqTLP29* protein sequence was

predicted using SignalP 4.1 Server (http://www.cbs.dtu.dk/services/SignalP/) and TMHMM Server v. 2.0 (http://www.cbs.dtu.dk/services/TMHMM/), which were both classified in CBS Prediction Servers. The nuclear localization signal in *VqTLP29* protein sequence was analyzed and predicted in cNLS Mapper (http://nls-mapper.iab.keio.ac.jp/cgi-bin/NLS_Mapper_form.cgi)[78].

Subcellular localization experiment of *VqTLP29*

The young leaves of tissue-cultured grape Red Globe seedlings were used for protoplast isolation. Grapevine mesophyll protoplast isolation was based on a protocol for the preparation of *Arabidopsia thaliana* and grape protoplasts with some modifications[79,80]. Leaves were sliced into 0.5-1.0 mm strips using a razor blade and transferred into 10 ml sterilized cell wall degrading enzyme buffer (20 mM MES, 4-morpholineethanesulfonic acid, pH 5.7; 0.4 M mannitol; 20 mM KCl; 10 mM $CaCl_2$; 0.1 % bovine serum albumin BSA, OSI Specialties, Sigma; 1.5 % cellulase R-10 and 0.4 % macerozyme R-10). A vacuum (0.07-0.08 MPa) was applied for 30 min and the infiltrated tissue strips were incubated in the dark for 12 h at 25℃. After incubation, an equal volume of W5 solution (2 mM MES, pH 5.7; 154 mM NaCl; 5 mM glucose; 125 mM $CaCl_2$ and 5 mM KCl) was added to stop the reaction. Protoplasts were filtered through a 75 lm nylon mesh and pelleted by centrifugation at room temperature (200 g, 3 min). After one wash with W5 solution, the pellets were resuspended in MMG solution (4 mM MES, pH 5.7; 0.4 M mannitol and 15 mM $MgCl_2$). The protoplast yield was measured immediately after purification under visible light using a hematocytometer to a concentration of $2\text{-}3\times10^6$.
For protoplast transfection assay, the binary plasmid PBI221-VqTLP29-GFP (20 μg) was added to above isolated protoplast solution (100 μl) with the plasmid PBI221-GFP as positive control. Then an equal volume of freshly prepared PEG-Ca^{2+} solution (40 % PEG 4000; 0.4 M mannitol and 100 mM $CaCl_2$) was added for incubation for 5 min at room temperature. 440 μl W5 solution was slowly added to stop the reaction. The mixed protoplasts were pelleted by centrifugation at room temperature (200 g, 2 min). The protoplasts were resuspended in W5 solution and incubated for 20-25 h at room temperature in the dark before examination by fluorescence microscopy. Finally, leaf mesophyll protoplasts were observed using a OLYMPUS confocal microscope FV1000

(Tokyo, Japan) with a ×20 and ×40 objective lens. GFP fluorescent signals were acquired using 488 nm excitation wavelengths, while chlorophyll autofluorescence was monitored using 633 nm excitation wavelengths. The experiment was independently repeated at least three times with consistent results.

References

78. Kosugi, S., Hasebe, M., Tomita, M. & Yanagawa, H. Systematic identification of yeast cell cycle-dependent nucleocytoplasmic shuttling proteins by prediction of composite motifs. *Proc. Natl. Acad. Sci*. **106**, 10171-10176(2009).

79. Yoo, S. D., Cho, Y. H. & Sheen, J. *Arabidopsis* mesophyll protoplasts: a versatile cell system for transient gene expression analysis. *Nat. Proto*. **2**, 1565-1572(2007).

80. Zhao, F. L. *et al*. A highly efficient grapevine mesophyll protoplast system for transient gene expression and the study of disease resistance proteins. *Plant Cell Tiss Organ Cult*. **125**, 43-57(2016).

Supplementary Figures and Tables

Supplementary Table S1. Primers of 33 *VvTLP* genes

Gene ID	Gene locus ID	Primer	
		Forward primer(5' to 3')	Reverse primer(5' to 3')
VvTLP1	GSVIVT01011706001	TGTGCTCTTCCCTCCTGG	TACTGCCTGTTCCCTATC
VvTLP2	GSVIVT01011705001	TGACTCTGGGTGGTTTGA	TTTGGTAGGGTTGTAGGTT
VvTLP3	GSVIVG01019835001	CCCTGGCACAACTGGAG	GGCAGAAGATAACTTCATAGTTGG
VvTLP4	GSVIVT00001105001	AAAACCATTGCTCCTACGC	CATTGGAAGTAGGATTGAAGGC
VvTLP5	GSVIVT00001104001	CCAACACCATTGCCGAGTA	GTGGAGCACGTAAACAAAC
VvTLP6	GSVIVG01019836001	ATCACCCTCTTCTTCACC	GCACCCTAGTAGTTTGGA
VvTLP7	GSVIVG01019838001	CGTTGTTCCTACACAGTTTGGG	CATCCTTAGGGTAGCTGTA
VvTLP8	GSVIVG01019840001	TCCTGGAGCCTGACTGTGAAC	GGGCAGAAGATAACATCATAGTTG
VvTLP9	GSVIVG01019841001	TGCAGCAACCTTCAACATC	GTGGCGGCCTTCACAT
VvTLP10	GSVIVT01019848001	CTCCTCAGCCTCCTCTTT	ACAGCTTCCAGGTCCATC
VvTLP11	GSVIVG01019849001	CTGCTTCATCTCCTCCATCCA	ACCGGCCACGACCACTG
VvTLP12	GSVIVT01024052001	GCAAATCCAGGACCATCTC	TACCCATCAACCAAGCTCAC
VvTLP13	GSVIVG01024050001	ACACTTCCCGTTCGTTTCT	CACTATCATCGGCAGGTTG
VvTLP14	GSVIVT00008703001	ATGCGTACATTCCTGACC	AACAGGGTGCAACTTTGA
VvTLP15	GSVIVG01018769001	ATTCCCTATCCCTCCCTACT	GAGGTTATAGCCGTCCACC
VvTLP16	GSVIVG01018767001	TTGAAGGTTGAGGGTGGT	CTCCGTTACAGGTGAAAGTG
VvTLP17	GSVIVT01024997001	TGGAGGGAAGGGTAGTTT	AAAGGGACAGTTGTTGACG
VvTLP18	GSVIVT01034131001	CATTCTGATGCGACCTCC	TATACTTGCCGTTGCTCC
VvTLP19	GSVIVG01033694001	TCTCTTGACGTTCCCGCT	GAATCCGTCCACCAGACTCA
VvTLP20	GSVIVT01022993001	ACGATGTCAGCCTTGTTG	CTCCTGGAATAGTTTGTAGC
VvTLP21	GSVIVT00008847001	CACTTTAGAAAAATCTGTTGGCTT	TTTCGATATTCCCTACAGGTGG
VvTLP22	GSVIVG01032051001	AGACCAGGTGAATCAGTAGA	ACGACCATCCCTCAGAAC
VvTLP23	GSVIVT01016504001	AGCCTTACATTTCTATGATGTGAGT	CCAGTGCAACAATACCTATCTGAT
VvTLP24	GSVIVG01032560001	AATGCAGCAGCACAACCG	AGCGACCATCACAGTCACCA

VvTLP25	GSVIVT01018147001	AGCTCCACCCTTCGGTAA	TTACAAGAAATTGAAGTGCAGAAGG
VvTLP26	GSVIVG01027712001	GGGGCCGCCGTAGCT	GAATATTGACACTGGCATGTTGTAT
VvTLP27	GSVIVT01027698001	CGATTGAATGGCGGTTTC	CCCAGGCTTTAGATGGTT
VvTLP28	GSVIVG01038679001	CTTTGTTTCTCATTGCCATTTC	TTCGACCCGACCATCCTG
VvTLP29	GSVIVG01008423001	AGGTGAGATCCCACGACAA	AGGGCAGAAAGTGACCAAAT
VvTLP30	GSVIVG01009646001	CGGCCACAGTGTTTACCC	CAGAACCTTCCCGACCAT
VvTLP31	GSVIVG01009928001	ACCAGGAGTGTTATCAGCAA	ATGAGGAAGGCACCGTTATG
VvTLP32	GSVIVG01009930001	TAGGCTGGTCAGGGAGGT	CCACGATCATGGGTAGGTT
VvTLP33	GSVIVT01008918001	TACTCCAAGAAGTTCAAGGAGG	TGTTGTTGTGGTAGGTGC

Supplementary Figure S1. Expression patterns of the 33 *VvTLP* genes.

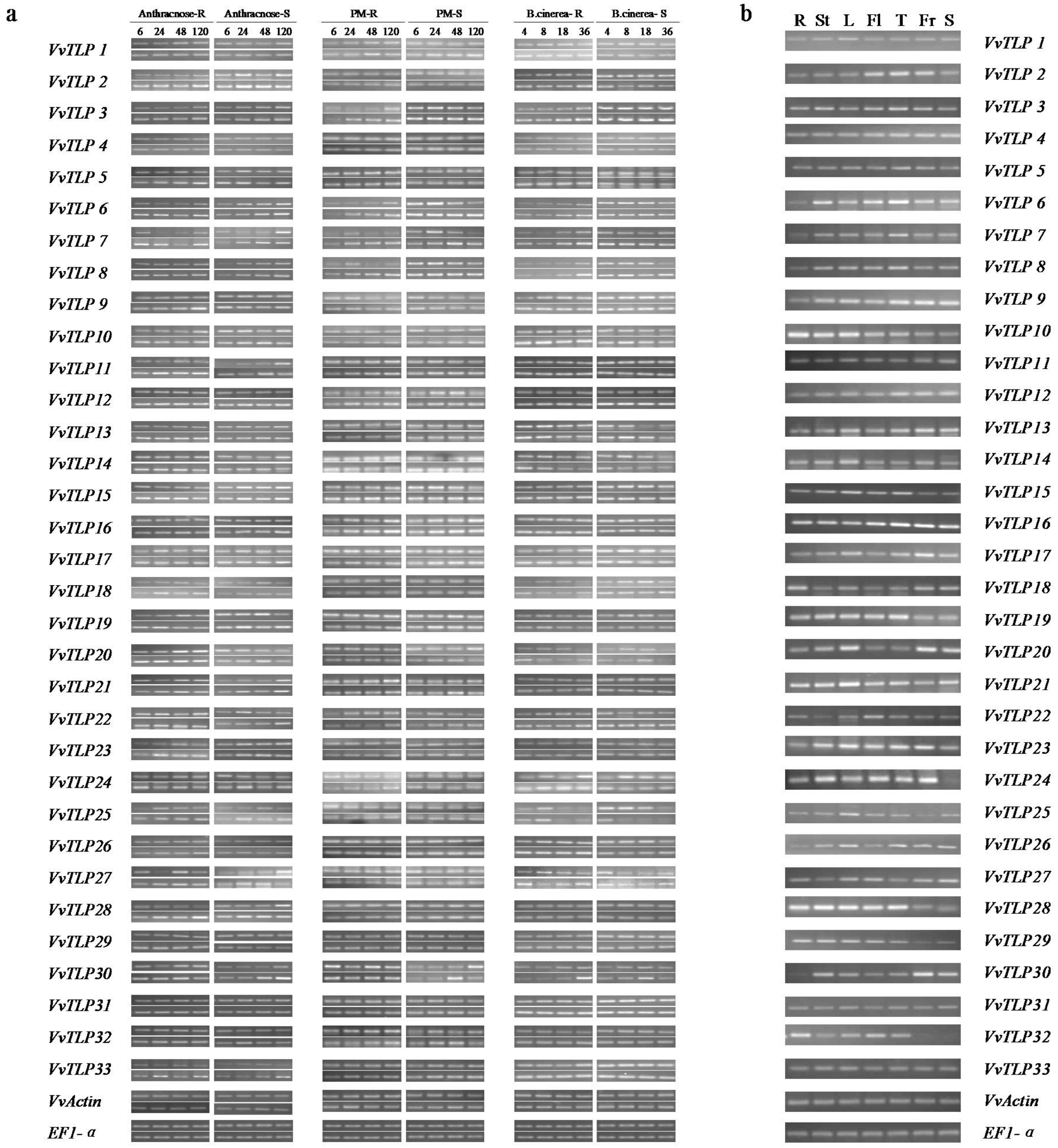

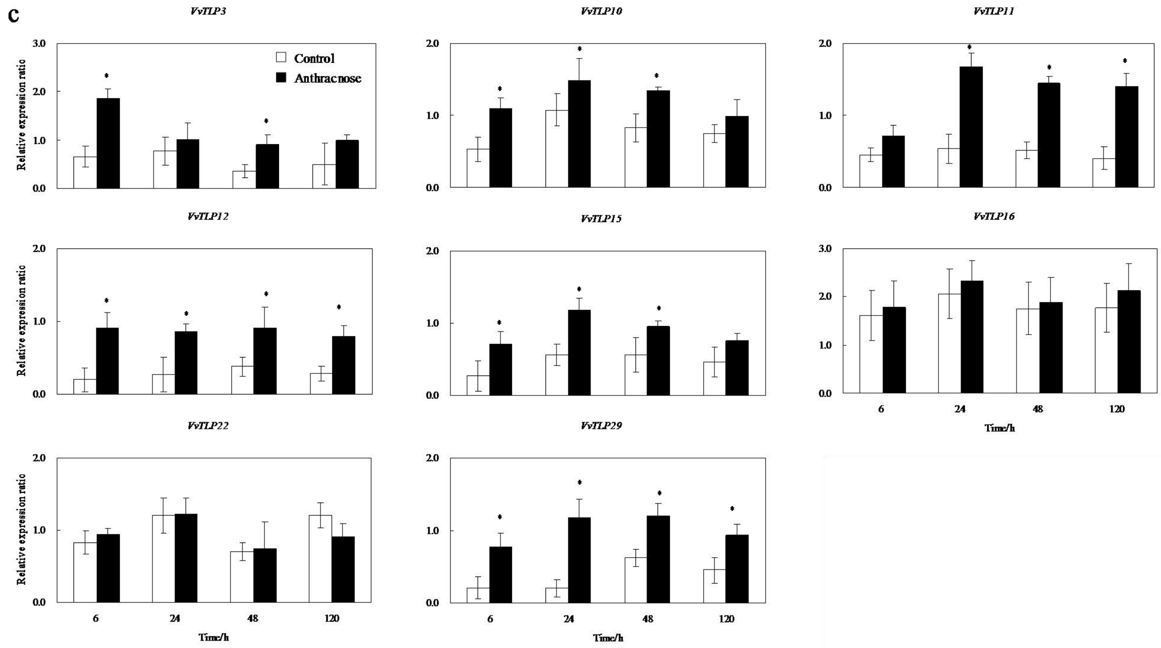

Supplementary Figure S1: a. Expression patterns of the 33 *VvTLP* genes after inoculation with different pathogens. Anthracnose-resistant (Anthracnose-R) grape 'Shang-24' (*V. quinquangularis*) and anthracnose-susceptible (Anthracnose-S) grape Red Globe (*V. vinifera*) were used for *Elsinoe ampelina* inoculation (left). Powdery mildew-resistant (PM-R) grape 'Shang-24' and powdery mildew-susceptible (PM-S) grape 'Hunan-1' (*V. pseudoreticulata*) were used for *Erysiphe necator* inoculation (middle). *Botrytis cinerea*-resistant (*B. cinerea*-R) grape 'Shuangyou' (*V. amurensis*) and *Botrytis cinerea*-susceptible (*B. cinerea*-S) grape Red Globe were used for *B. cinerea* inoculation (right). For each *VvTLP* gene, two electrophoretic bands are shown, the upper one being the control and the lower one being the expression after treatment. b. Expression patterns of 33 *VvTLP* genes in different organs of 'Shang-24'. R: root, St: stem, L: leaf, Fl: flower, T: tendril, Fr: fruit, S: seed. c. Eight genes (*VvTLP3*, *VvTLP10*, *VvTLP11*, *VvTLP12*, *VvTLP15*, *VvTLP16*, *VvTLP22* and *VvTLP29*) were selected with thaumatin domain from Type I and II subfamily for further validation of their expression patterns in 'Shang-24' infected with anthracnose in three independent experiments with consistent results. The data are means ± SD from triplicate experiments with consistent results. Asterisks indicate statistical significance (*: $P < 0.05$, one-way ANOVA).

Supplementary Data S1. Analysis of *TLP29* sequence homology.

```
GSVIVT01008423001    ATGGGGATGCTGCTCAGGTCCCTGCTTCTCAGTCTCGTCTGCACCTGCATTGCGGGTGAAGTATCGGCCACAACAATTTCACTATACAACAAATGCTCAC    100
VqTLP29              ATGGGGATGCTGCTCAGGCCCCTGCTTCTCAGTCTCGTCTGCACCTACATTGCGGGTGAAGTATCGGCCACAACAATTTCACTATACAACAAATGCTCAC    100
VvTLP29              ATGGGGATGCTGCTCAGGTCCCTGCTTCTCAGTCTCGTCTGCACCTACATTGCGGGTGAAGTATCGGCCACAACAATTTCACTATACAACAAATGCTCAC    100

GSVIVT01008423001    ATCCAGTGTGGCCAGGCATACAACCAGGCGCAGGCAAGCCCATCCTTGCCCGTGGAGGCTTCAAGCTCCCACCCAACAAGGCCTACTCCCTCCACATCCC    200
VqTLP29              ATCCAGTGTGGCCAGGCATACAACCAGGCGCAGGCAAGCCCATCCTTGCCCGTGGAGGCTTCAAGCTCCCACCCAACAAGGCCTACTCCCTCCACATCCC    200
VvTLP29              ATCCCGTGTGGCCAGGCATACAACCAGGCGCAGGCAAGCCCATCCTTGCCCGTGGAGGCTTCAAGCTCCCACCCAACAAGGCCTACTCCCTCCACATCCC    200

GSVIVT01008423001    AGCGGCCTGGTCCGGCCGCATCTGGGGCCGTCACGGATGCTCCTTCGACGCCCACGGCCGGGGCCGCTGCGCTACCGGCGACTGCGGCGGCTCCCTTTTC    300
VqTLP29              AGCGGCCTGGTCCGGCCGCATCTGGGGCCGTCACGGCTGCTCCTTCGACGCCCATGGCCGGGGCCGCTGCGCTACCGGCGACTGCGGCGGCTCCCTTTTC    300
VvTLP29              AGCCGCCTGGTCCGGCCGCATCTGGGGCCGTCACGGATGCTCCTTCGACGCCCACGGCCGGGGCCGCTGCGCTACCGGCGACTGCGGCGGCTCCCTTTTC    300

GSVIVT01008423001    TGCAACGGCATGGGCGGCACACCTCCGGCCACCCTCGCCGAGATCACCCTGGGTTCGGAGCAAGATTTCTACGACGTGAGCCTCGTCGACGGTTACAACC    400
VqTLP29              TGCAACGGCATGGGCGGCACACCTCCGGCCACCCTCGCCGAGATCACCCTGGGTTCGGAGCAAGATTTCTACGACGTGAGCCTCGTCGACGGTTACAACC    400
VvTLP29              TGCAACGGCATGGGCGGCACACCTCCGGCCACCCTCGCCGAGATCACCCTGGGTTCGGAGCAAGATTTCTACGACGTGAGCCTCGTCGACGGTTACAACC    400

GSVIVT01008423001    TGGCCATCTCCATCACCCCCTTCAAAGGGTCAGGCAAGTGTAGCTACGCGGGATGTGTGAGCGACCTCAACACCATGTGTCCGGTGGGTCTCCAGGTGAG    500
VqTLP29              TGGCCATCTCCATCACCCCCTTCAAAGGGTCAGGCAAGTGTAGCTACGCGGGATGTGTGAGCGACCTCAACACCATGTGTCCGGTGGGTCTCCAAGTGAG    500
VvTLP29              TGGCCATCTCCATCACCCCCTTCAAAGGGTCAGGCAAGTGTAGCTACGCGGGATGTGTGAGCGACCTCAACACCATGTGTCCGGTGGGTCTCCAGGTGAG    500

GSVIVT01008423001    ATCCCACGACAACAGGCGGGTGGTGGCCTGCAAGAGCGCATGCTCTGCCTTCAACTCGCCCCGCTACTGCTGCACTGGAAGCTTTGGGAGTCCGCAGTCC    600
VqTLP29              ATCCCACGACAACAGGCGGGTGGTGGCCTGCAAGAGCGCATGCTCTGCCTTCAACTCGCCCCGCTACTGCTGCACTGGAAGCTTTGGGAGTCCGCAGTCC    600
VvTLP29              ATCCCACGACAACAGGCGGGTGGTGGCCTGCAAGAGCGCATGCTCTGCCTTCAACTCGCCCCGCTACTGCTGCACTGGAAGCTTTGGGAGTCCGCAGTCC    600

GSVIVT01008423001    TGCAAGCCCACCGCCTACTCCAAGATCTTCAAAGCCGCATGTCCAAGAGCTTATTCCTATGCTTATGATGATCCAACTAGCATTGCCACTTGCACTGGTG    700
VqTLP29              TGCAAGCCCACCGCCTACTCCAAGATCTTCAAAGCCGCATGTCCAAGAGCTTATTCCTATGCTTATGATGATCCAACTAGCATTGCCACTTGCACTGGTG    700
VvTLP29              TGCAAGCCCACCGCCTACTCCAAGATCTTCAAAGCCGCATGTCCAAGAGCTTATTCCTATGCTTATGATGATCCAACTAGCATTGCCACTTGCACTGGTG    700

GSVIVT01008423001    GCAACTATTTGGTCACTTTCTGCCCTCACCACTAG                                                                     735
VqTLP29              GCAACTATTTGGTCACTTTCTGCCCTCACCACTAG                                                                     735
VvTLP29              GCAACTATTTGGTCACTTTCTGCCCTCACCACTAG                                                                     735
```

Supplementary Data S1: 99.32% and 99.58% *TLP29* sequence homology to *V. vinifera* (GSVIVT01008423001, grape genomic sequence) from that of *V. quinquangularis* cv. ‘Shang-24’ and *V. vinifera* cv. Red Globe. Sequence homology colored with dark blue was 100% and that with light blue was over 75%.

Supplementary Data S2. Analysis of *TLP29* protein sequence alignment.

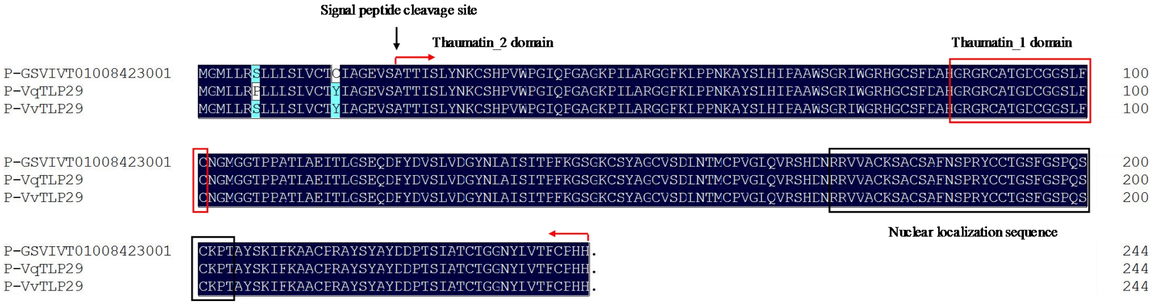

Supplementary Data S2: *TLP29* protein sequence alignment from that of *V. quinquangularis* cv. 'Shang-24' and *V. vinifera* cv. Red Globe to *V. vinifera* (GSVIVT01008423001). The sequence in red box represents Thaumatin_1 domain and the sequence indicated by the red arrows represents Thaumatin_2 domain using the PROSITE; the amino acid site targeted with black arrow represents the signal peptide cleavage site in *TLP29* protein sequence using CBS Prediction Servers (TMHMM Server v. 2.0 and SignalP 4.1 Server) on line; the sequence in black box represents the nuclear localization sequence assessed as three points using the cNLS Mapper on line; '.' represents stop codon. Sequence homology colored with dark blue was 100% and that with light blue was over 75%.

Supplementary Figure S2. Expression analysis of *VqTLP29* in three transgenic *Arabidopsis thaliana* lines and Col-0 following different pathogens inoculation.

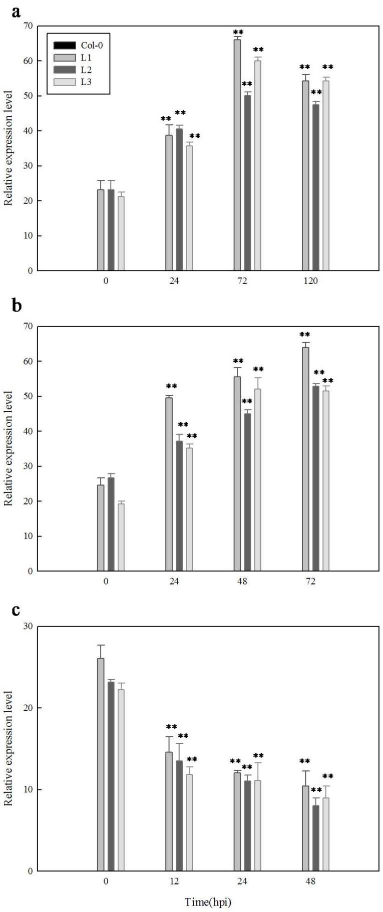

Supplementary Figure S2: Real-time quantitative PCR for expression patterns of *VqTLP29*. a. The *VqTLP29* expression levels following powdery mildew (*Golovinomyces cichoracearum*, UCSC1) inoculation in transgenic *A. thaliana* lines L1, L2, L3 and Col-0. Compared to the expression level at 0 hpi, the *VqTLP29* expression levels gradually increased and peaked at 72 hpi. b. The expression levels of *VqTLP29* following DC3000 inoculation. The *VqTLP29* expression levels gradually increased and peaked at 72 hpi. c. The expression levels of *VqTLP29* following *B. cinerea* inoculation. *VqTLP29* expression levels gradually decreased to the minimum value at 48 hpi. The *VqTLP29* transcript levels were 0 in Col-0. The relative expressions of *VqTLP29* were analyzed based on the *AtActin* expression levels. The data are means ± SD from triplicate experiments with consistent results. Asterisks indicate statistical significance (**: $P < 0.01$, one-way ANOVA).

Supplementary Table S2. Primers of resistance-related genes in *Arabidopsis thaliana*

Gene ID	Gene locus TAG	Primer	
		Forward primer(5' to 3')	Reverse primer(5' to 3')
PR1	AT2G14610	AACTACGCTGCGAACACGTG	TCACTTTGGCACATCCGAGTC
NPR1	AT1G64280	ACATCACCGGGTGTAAAGAT	AAGCCAGTTGAGTCAAGTCC
ICS1	AT1G74710	CTTCCGTGACCTTGATCCTTTCT	CAGCGATCTTGCCATTAGGATC
WRKY53	AT4G23810	GCGACAAGACACCAGAGTCAA	CGCCGTTGATAGTTCCGTAAT
NHL10	AT2G35980	TTCCTGTCCGTAACCCAAAC	CCCTCGTAGTAGGCATGAGC
FRK1	AT2G19190	GCCAACGGAGACATTAGAG	CCATAACGACCTGACTCATC
LOX3	AT2G35980	TCTCCGTACAACAAGCGTTGG	GCGTCCGTCTAGCGCATTAAT
PDF1.2	AT5G44420	GAAGCACAGAAGTTGTGCGA	TGTAACAACAACGGGAAAATAAACA
COI1	AT2G39940	ATTGATTCCGAGACGAGA	AACCCAAGGAGTAACATAAC
FLS2	AT5G46330	ACCAATACCTTCCAGCAT	CCTCCCAATAGAAATGAAC
OST1	AT4G33950	ATGGATCGACCAGCAGTG	GGTTGATTATCTCCCTTT
ATPPC2	AT2G42600	TGCGGTGACAGACCTATT	ACCTAAGTGCGTCGTGAT
AtActin	AT2G37620	GGTAACATTGTGCTCAGTGGTGG	AACGACCTTAATCTTCATGCTGC

Supplementary Figure S3. Characterization of the *VqTLP29* over-expressing *Arabidopsis thaliana* lines.

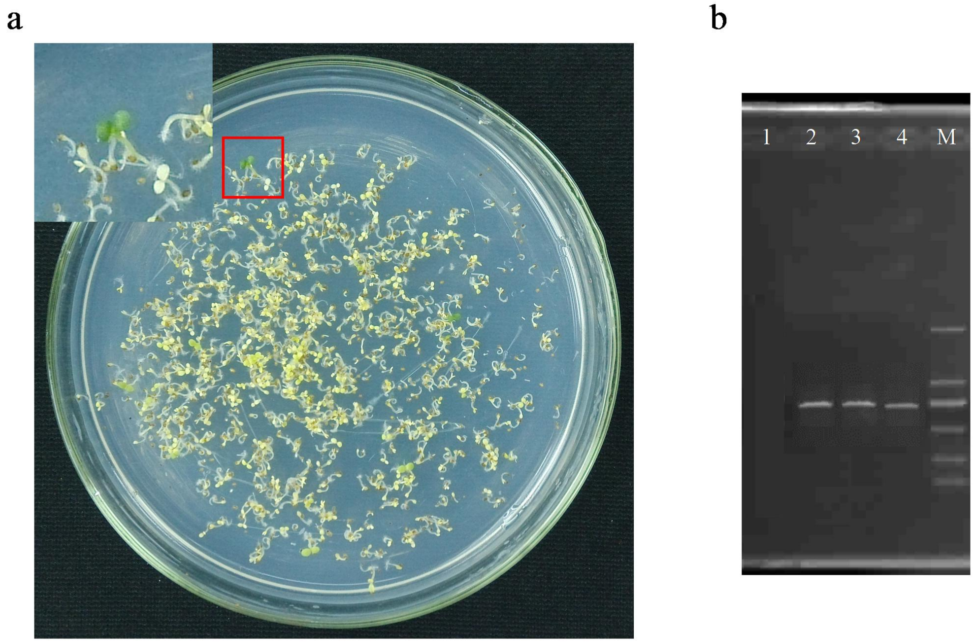

Supplementary Figure S3: a. Screening of kanamycin-resistant *VqTLP29* transgenic *Arabidopsis* seedlings in T1 generation using MS medium contained 60 mg L^{-1} kanamycin. b. Identification of *VqTLP29* in three transgenic line L1, L2, L3 and Col-0 in DNA level. Lane 1: Col-0; lane 2-4: three transgenic lines; M: DNA Maker 2000. Electrophoretic bands represent 735 bp.

Supplementary Figure S4. Subcellular localization of *VqTLP29* in grape mesophyll protoplasts.

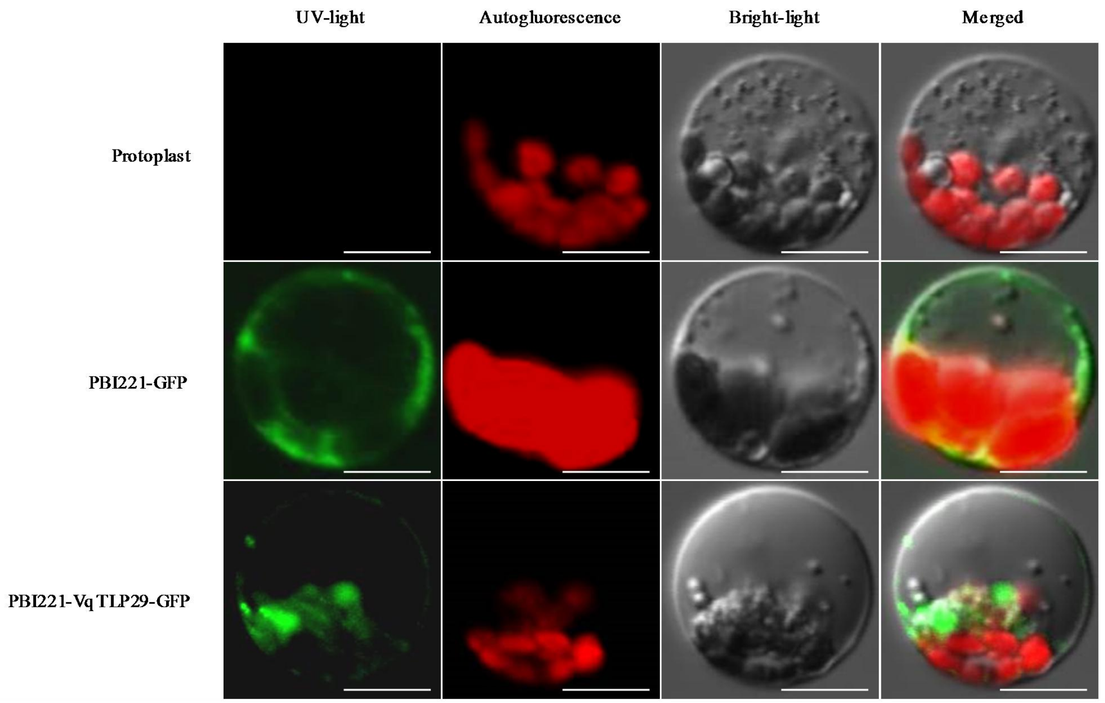

Supplementary Figure S4. The schematic illustrations in grape mesophyll protoplasts with protoplasts under normal condition (up, as negative control), the vectors PBI221-GFP (middle, as positive control) and PBI221-VqTLP29-GFP (down) photographed with a OLYMPUS confocal microscope FV1000 and a ×20 objective lens. The first vertical columns shown green filled boxed indicate green fluorescence protein (GFP) using UV-light under 488 nm excitation wavelengths. The second vertical columns shown red filled boxed indicate chlorophyll autofluorescence under 633 nm excitation wavelengths. The third vertical columns indicate bright field images of protoplasts using bright-light. The forth vertical columns indicate the merged images of GFP, autofluorescence and bright-light using OLYMPUS FLUOVIEW Ver 3.1 software of the OLYMPUS confocal microscope. The result showed that *VqTLP29* protein as a secerted protein was located in the cytoplasm shown in the merge image. The experiments were repeated three times with consistent results. The scale bar in the figure indicates 10 μm.

Supplementary Table S3. Percentages of efficiency of grape mesophyll protoplasts transfection.

	Percentages of the protoplast transfection (%)			Average of the percentages (%)
	Test 1	Test 2	Test 3	
Protoplast	—	—	—	—
PBI221-GFP.	53.6	36.0	39.3	43.0
PBI221-VqTLP29-GFP	33.3	23.1	26.9	27.8*

Supplementary Table S3. Percentages of the protoplast transfection represent the ratios of fluorescent cells numbers to total cells numbers; Average of percentages represents the average numbers of three tests; * represents the statistical significance with 30 protoplasts per test (*: $0.01 < P < 0.05$, one-way ANOVA). The concentration of protoplasts (2-3 $\times 10^6$), plasmid concentration (20 μg) and PEG incubation time (5 min) were the pivotal factors in the three experiments of subcellular localization of *VqTLP29*. The result showed that no green fluorescence was identifiable in the non-transfected protoplasts and the transfection efficiencies of PBI221-VqTLP29-GFP and PBI221-GFP were 27.8% and 43.0%, which were both relatively low. And the transfection efficiency of PBI221-VqTLP29-GFP was significantly lower than that of PBI221-GFP.
